# Long-term historical and projected herbivore population dynamics in Ngorongoro crater, Tanzania

**DOI:** 10.1371/journal.pone.0212530

**Published:** 2020-03-10

**Authors:** Patricia D. Moehlman, Joseph O. Ogutu, Hans-Peter Piepho, Victor A. Runyoro, Michael B. Coughenour, Randall B. Boone

**Affiliations:** 1 IUCN/SSC Equid Specialist Group, Arusha, Arusha, Tanzania; 2 Institute for Crop Science-340, University of Hohenheim, Stuttgart, Germany; 3 Environmental Reliance Consultants Limited, Arusha, Tanzania; 4 Department of Ecosystem Science and Sustainability, Colorado State University, Fort Collins, Colorado, United States of America; 5 Natural Resource Ecology Laboratory, Colorado State University, Fort Collins, Colorado, United States of America; Texas State University, UNITED STATES

## Abstract

The Ngorongoro Crater is an intact caldera with an area of approximately 310 km^2^ located within the Ngorongoro Conservation Area (NCA) in northern Tanzania. It is known for the abundance and diversity of its wildlife and is a UNESCO World Heritage Site and an International Biosphere Reserve. Long term records (1963–2012) on herbivore populations, vegetation and rainfall made it possible to analyze historic and project future herbivore population dynamics. NCA was established as a multiple use area in 1959. In 1974 there was a perturbation in that resident Maasai and their livestock were removed from the Ngorongoro Crater. Thus, their pasture management that was a combination of livestock grazing and fire was also removed and 'burning' stopped being a regular occurrence until it was resumed in 2001 by NCA management. The Maasai pasture management would have selected for shorter grasses and more palatable species. Vegetation mapping in 1966–1967 recorded predominately short grasslands. Subsequent vegetation mapping in the crater in 1995 determined that the grassland structure had changed such that mid and tall grasses were dominant. After removal of the Maasai pastoralists from the Ngorongoro Crater in 1974, there were significant changes in population trends for some herbivore species. Buffalo, elephant and ostrich numbers increased significantly during 1974–2012. The zebra population was stable from 1963 to 2012 whereas population numbers of five species declined substantially between 1974 and 2012 relative to their peak numbers during 1974–1976. Grant’s and Thomson’s gazelles, eland, kongoni, and waterbuck (wet season only) declined significantly in the Crater in both seasons after 1974. In addition, some herbivore species were consistently more abundant inside the Crater during the wet than the dry season. This pattern was most evident for the large herbivore species requiring bulk forage, i.e., buffalo, eland, and elephant. Even with a change in grassland structure, total herbivore biomass remained relatively stable from 1963 to 2012, implying that the crater has a stable carrying capacity. Analyses of rainfall indicated that there was a persistent cycle of 4.83 years for the annual component. Herbivore population size was correlated with rainfall in both the wet and dry seasons. The relationships established between the time series of historic animal counts in the wet and dry seasons and lagged wet and dry season rainfall series were used to forecast the likely future trajectories of the wet and dry season population size for each species under three alternative climate change scenarios.

## Introduction

Biodiversity conservation is facing widespread and mounting challenges world-wide primarily due to human population expansion, land use changes, overexploitation of biodiversity, climate change, invasive species and their interactions, leading to unprecedented biodiversity losses [1]. Effective biodiversity conservation therefore urgently requires an accurate understanding of biodiversity dynamics and their primary drivers as a basis for developing sound conservation policies and management strategies. Rainfall primarily governs vegetation production and quality, surface water availability and quality in savannas [2–6] and therefore also the aggregate and species-specific biomass levels of large African savanna ungulates [7–9]. Consequently, it is important to first establish the influence of rainfall variation on large herbivore population dynamics before the contributions of other factors can be reliably established [10].

In the Serengeti-Mara ecosystem straddling Kenya and Tanzania, rainfall primarily drives population dynamics [11,12], aggregate population biomass [7, 8], recruitment dynamics [13], phenology, synchrony and prolificacy of calving [14,15,16], seasonal dispersal and migration of large herbivores [17,18]. However, increasing frequency and intensity of droughts [19,20], widening variation of river flows [21] and rising temperatures [19] hasten the need to advance our understanding of how anticipated climate changes will likely affect larger herbivore populations. Such understanding can be gained by studying rainfall influences on large herbivore population dynamics using long-term monitoring data from protected areas with relatively little human influence, such as the Ngorongoro Crater, in northern Tanzania.

The Ngorongoro Crater in Tanzania is known worldwide for the abundance and diversity of its wildlife [22]. It is situated in the Crater Highlands and is linked both to this area and the Serengeti Plains by the seasonal migration of several herbivores [22–24] and the emigration and immigration of large carnivores [25–29].

Earlier analyzes of large herbivore population dynamics in the crater [23,30,31] have described temporal trends but not formally related them to their putative drivers. Earlier studies have identified droughts, disease, predation and their interactions and poaching as the main drivers of large herbivore population change in the crater.

Severe drought years combined with disease can adversely impact herbivore populations. For example, in 2000 and 2001 there was significant mortality in buffalo (1500 animals), wildebeest (250) and zebra (100) apparently due to nutritional stress resulting from the severe drought in the dry season in 2000 [23,32,33].

Poaching was the major impact on the Black rhino population from the 1970’s to mid-1980’s. Since the early 1990's there has been limited poaching and the population is slowly recovering.

Conservative population projections in 1995 [34] predicted that in the best scenario, i.e., no poaching, the population should be approximately 35 to 40 individuals by 2017. The Black rhino population was 59 individuals in 2018 (Pers comm, M. Musuha, 2018, NCAA).

Since 1963, the herbivore population of Ngorongoro Crater has been monitored by the Ngorongoro Conservation Area Authority (NCAA), The College of African Wildlife Management and research scientists [23–24,30,35–37]. Since 1978, the Ngorongoro Ecological Monitoring Program has been responsible for conducting the wet and dry season systematic ground counts. The complete data set covers a period of 50 years (1963–2012).

Here, we analyze the long-term population trends of the 12 most common large herbivore species as well as the aggregate biomass of the multi-species wild herbivore community and relate the trends quantitatively to historic rainfall and projected rainfall variation under three climate change scenarios and qualitatively to vegetation changes and burning. Relationships established between historic population abundance and historic rainfall are used to project the impacts of three different future rainfall scenarios on wild herbivore population dynamics to 2100.

In particular, we test predictions of the following hypotheses. First, the eviction of Maasai, the removal of their livestock and cessation of grassland burning, resulted in changes in rangeland management that generated complex changes in vegetation composition and structure and hence in wild herbivore population numbers [23,30,31]. Pastoralists used livestock grazing and fire to manage grass structure and forage quality [30,31,38–41].

Second, the removal of the Maasai and their range management (livestock grazing and burning) from the crater in 1974 affected the plant structure in the crater and the population dynamics of the resident wild herbivore species depending on their life-history traits (body size, gut morphology) and life-history strategies (feeding style, foraging style, and movement patterns).

Third, rainfall variation influences the herbivore population dynamics and density, differentiated by life-history traits and strategies. Extreme rainfall in the crater, which waterlogs large parts of the crater, could adversely affect wildlife, just like droughts, if large parts of the crater become waterlogged. Additionally, high rainfall promotes grass growth and dilutes plant nutrients, hence reducing vegetation quality for herbivores.

Fourth, the projected ungulate population dynamics should mirror the pronounced and sustained oscillations in future rainfall projected under each of three climate change scenarios (S5 Fig). Further, large-sized herbivores dependent on bulk, low-quality forage should prosper under the wet and cooler conditions expected under the best case (RCP 2.6) scenario (S5 Fig). Likewise, small-sized herbivores requiring high-quality forage should thrive under the relatively low rainfall and warmer conditions anticipated under the intermediate (RCP 4.5) and worst-case (RCP 8.5) scenarios. The warmer temperatures expected under RCP 8.5 than under RCP 4.5 imply that conditions should be most arid and therefore most stressful to water dependent herbivores under this scenario.

## Methods

### Study area

**Fig 1:**
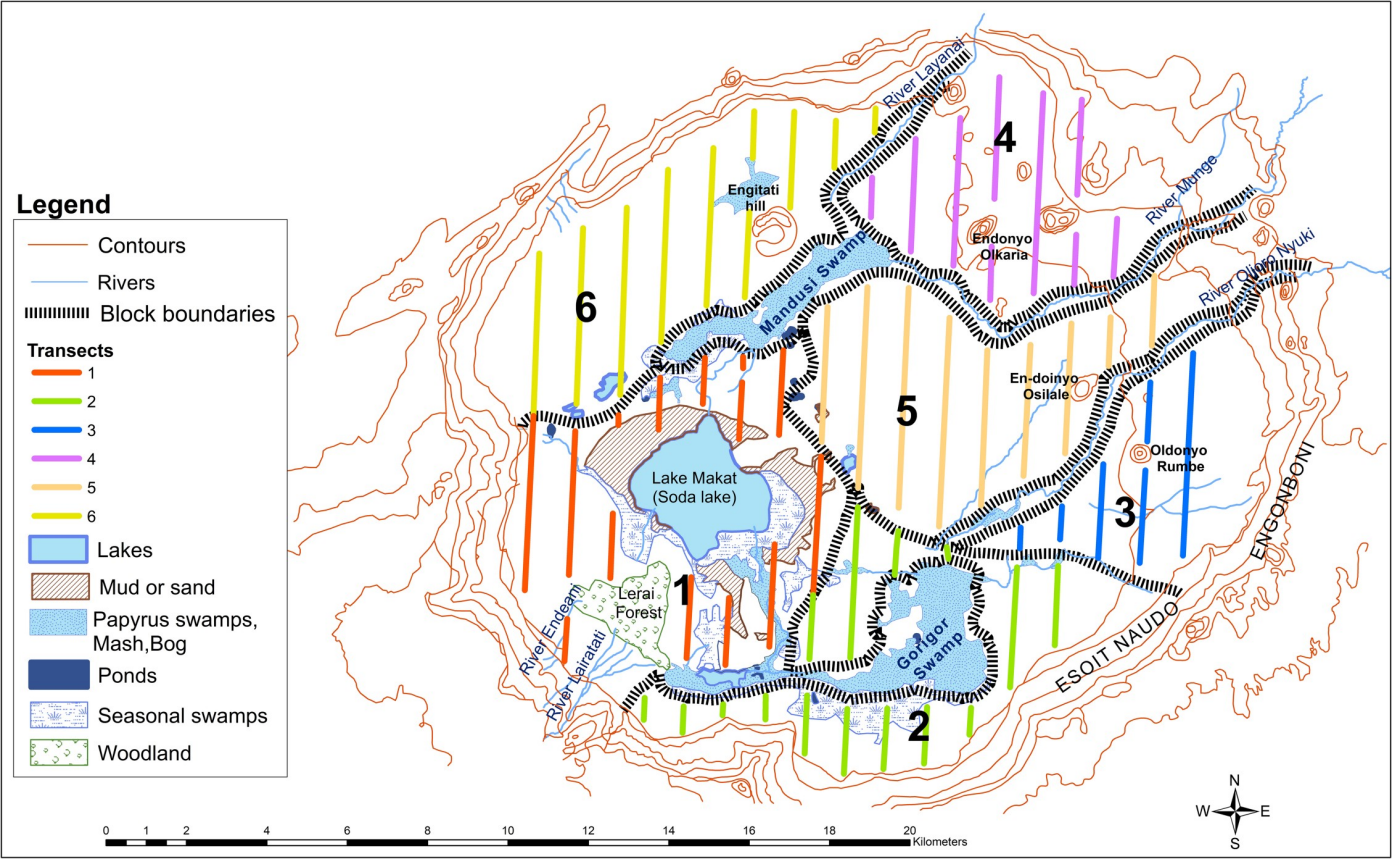
Ngorongoro crater and census blocks [12]).

Ngorongoro Crater, Tanzania, is known worldwide for the abundance and diversity of its wildlife [22,23,30,31]. The crater (3°10′ S, 35° 35′ E) is a large intact caldera with an area of approximately 310 km^2^. The floor of the crater is about 264 km^2^ (1,800 m above sea level) and the sides rise steeply 500 meters to the rim. The geology, soils and vegetation of the crater were described by Herlocker and Dirschl [42] and Anderson and Herlocker [43]. The crater has the largest catchment basin in the Ngorongoro Highlands [44] and receives water from Lalratati and Edeani streams and Lerai spring from Oldeani Mountain to the south. Seneto spring provides water to Seneto swamp and Lake Magadi from the southwest. Olmoti Crater provides runoff to Laawanay and Lemunga rivers in the north, which supply Mandusi swamp and Lake Magadi. Lljoro Nyuki river, in the northeast provides water to Gorigor swamp. Ngaitokitok spring in the eastern part of the crater also supplies Gorigor swamp and Lake Magadi. Soil characteristics and drainage affect vegetation species and during the dry season soil moisture is dependent on the crater’s catchment system (Fig 1). The wildlife of Ngorongoro Crater has had a protected status since 1921 [30]. The area has been administered by the Ngorongoro Conservation Unit since 1959 and by the Ngorongoro Conservation Area Authority since 1975 as part of a protected multiple land use area (8,292 km^2^). Maasai pastoralists are believed to have inhabited the Ngorongoro Crater since the mid 1800's when they displaced the Datoga people [33]. In 1974, resident Maasai pastoralists, their bomas and livestock were removed from the crater [36,45]. This meant that their pasture management, which was a combination of livestock grazing and fire ceased and potentially vegetation structure, species composition and forage quality were affected.

### Vegetation

Changes in vegetation composition and structure were measured by digitizing and comparing vegetation maps that were done in 1966–67 and 1995 [42,46]. The Herlocker and Dirschl map of the crater was based on aerial photographs and stereo interpretations of mapping units and the grasslands were divided into three height classes. The vegetation of the final mapping units was then sampled to determine structure and species composition [42]. The Chuwa and Moehlman map was based on the Ngorongoro 1:50,000 map issued by the Government of the United Republic of Tanzania Surveys and Mapping Division in 1975. Transects were driven to identify vegetation units and a sliding point frame was used to measure species composition. Grass leaf height was measured at the mid-point of the frame [46]. Maps were digitized in ArcGIS 9.1 (ESRI, Redlands, California) and projected to UTM Zone 36, WGS 1984 datum. Attributes on the maps were digitized, and in both maps the plant height for primary and secondary canopy species was used to determine the presence of short, mid, mid-tall and tall grass structure. The vegetation composition and structure map of the crater has not been updated since 1995.

A comparison of the vegetation maps from before and after the removal of pastoralists and their livestock indicate that major changes occurred in vegetation structure.

From 1966/67 [42] to 1995 [46] there have been significant changes in the structure of the major and secondary herbaceous species. In 1966/67 the crater floor was dominated by short grass herbaceous species. By 1995, most of the short grasslands had been replaced by mid to tall plant species (S15 Table). Maasai pastoralists manage their grazing areas with movement of livestock and fire [38,39, 44]. This type of range management selects for shorter grasses and more palatable species [40,41] and was largely responsible for the decrease in the availability of short grasses and increase in medium and tall grasslands in the crater floor by 1995.

Fires were suppressed from 1974, when the Maasai were removed, until 2001 [32]. Prescribed burning by the NCAA management resumed in 2001. From 2002 to 2011 there was prescribed burning but no records were maintained. Trollope [38,39] recommended that areas with more than 4000 kg/ha should be burned every year at the end of the dry season (September/October) and that 10–20% of the crater floor be burned on a rotational basis. Highest tick density occurred in the peak dry season (September-October) in the tallest grass.

### Disease

Disease has played a role in the herbivore population trends in the crater. The Ngorongoro Conservation Area Authority (NCAA) started an inoculation campaign against rinderpest in the 1950’s and eradicated the disease by the 1960’s [47]. Inoculations against rinderpest for cattle continued but there was an outbreak in 1982 that affected buffalo and eland but not cattle [48]. Despite the losses from rinderpest during 1982, the buffalo population increased steadily from 1980 and had doubled by 1987.

Parasites and disease, specifically high tick burdens and tick-borne protozoan diseases, likely contributed to buffalo mortality in the crater in 2000 and 2001 following the 2000 drought [33]. Lion necropsy’s revealed the presence of tick-borne parasites (*Ehrlichia spp*., *Babesia* and *Theileria sp*) but canine distemper and a plague of *stomoxys* stinging flies were also implicated in lion mortality in the crater in 2000 and 2001 but the major cause of mortality was not conclusively determined [32].

Disease also had an impact on Black rhinos (*Diceros bicornis*) in the crater. In 2001, reports indicated that three Black rhinos died from a novel *Babesiosis bicornis* ap.nov. which may have been introduced by Black rhinos translocated from Addo Elephant National Park, South Africa. The impact of the novel parasite may have been exacerbated by drought and high tick densities [33]. The remaining 10 Black rhinos were treated with a curative babesicidal drug and survived [32].

The transmission of disease has been correlated with tick vector densities. Prescribed burning was started in the dry season of 2001 and 27 months later there was a significant difference between burned and unburned areas, with almost no adult ticks and relatively few immature ticks in the burned areas [34].

### Study populations

Long term data sets were available for 11 mammalian herbivore species (S2 Data), i.e. Wildebeest (*Connochaetes taurinus*), Plains Zebra (*Equus quagga)*, Cape Buffalo (*Syncerus caffer*), Thomson's gazelle (*Eudorcas thomsonii*), Grant's gazelle (*Nanger granti*), Eland (*Taurotragus oryx*), Kongoni (*Alcelaphus buselaphus cokii*), Waterbuck (*Kobus ellipsiprymnus*), Warthog (*Phacochoerus africanus)*, Elephant (*Loxodonta africana*) and Black rhino and one bird, the Ostrich (*Struthio camelus*).

Zebra are mid-sized herbivores, but they are non-ruminants. Hence, they are not limited by a four-chambered stomach system and can opt to consume larger amounts of higher fiber (lower quality) grasses to meet their nutritional requirements [49]. Elephant, Black rhino and warthog are non-ruminants.

Buffalo are large bodied ruminants and although they require a larger amount of food per individual, the quality can be lower and they can tolerate a higher proportion of fiber in their diet [49,50]. Buffalo prefer longer grass and select for a high ratio of leaf to stem [51]. Thomson’s gazelle, Grant’s gazelle, kongoni, wildebeest, waterbuck, and eland are small to mid-sized ruminants and have less tolerance for high-fiber forage [49].

The herbivores were classified into functional categories, i.e., grazers (Thomson’s gazelle, kongoni, wildebeest, eland, buffalo, and zebra) and mixed browsers/grazers (Grant’s gazelle, waterbuck, Black rhino and elephant) [52–58]. The ostrich is primarily an herbivore, but will also eat invertebrates and occasionally rodents [59].

### Rainfall

Long-term rainfall data were not available for the crater floor. We therefore used monthly rainfall measured from 1964 to 2014 at Ngorongoro Headquarters on the southern rim of the crater. The rainfall recorded at the Ngorongoro Conservation Area Authority (NCAA) Headquarters during 1963–2014 is provided in S1 Data.

### Projection of rainfall and temperature

Total monthly rainfall and average monthly minimum and maximum temperatures for Ngorongoro Crater were projected over the period 2013–2100 based on regional downscaled climate model data sets from the Coordinated Regional Climate Downscaling Experiment (CORDEX). Downscaling is done using multiple regional climate models as well as statistical downscaling techniques [60,61]. Three climate scenarios defined in terms of Representative Concentration Pathways (RCPs) were used to project rainfall and temperatures for the Ngorongoro Crater. The three RCPs are RCP 2.6, RCP 4.5 and RCP 8.5 in which the numeric suffixes denote radiative forcings (global energy imbalances), measured in watts/m^2^, by the year 2100. The RCP 2.6 emission pathway (best case scenario) is representative for scenarios leading to very low greenhouse gas concentration levels [62]. RCP 4.5 (intermediate scenario) is a stabilization scenario for which the total radiative forcing is stabilized before 2100 by employment of a range of technologies and strategies for reducing greenhouse gas emissions [63]. RCP 8.5 (worst case scenario) is characterized by increasing greenhouse gas emission over time representative for scenarios leading to high greenhouse gas concentration levels [64]. Rainfall, minimum and maximum temperature projections were made for a 50 × 50 km box defined by longitudes (34.97, 35.7) and latitudes (-3.38, -2.787).

### Herbivore systematic ground counts

Since 1963, the herbivore population of Ngorongoro Crater has been monitored by the Ngorongoro Conservation Area Authority, The College of African Wildlife Management and research scientists [22–24,30,35–37]. Since 1987, the Ngorongoro Ecological Monitoring Program has been responsible for conducting the wet and dry season censuses. This data set makes it possible to assess long-term population trends and the stability of this multi-species wild herbivore community. Here, we consider the data set (S2 Data) covering a period of 50 years (1963–2012).

Ground counts of large mammals in the wet and dry seasons have been done in the 264 km^2^ bottom of the crater since 1963. The floor of the crater was divided into six blocks of approximately 40 km^2^ each using ground features (Fig 1) that cover the entire area except for inaccessible areas, i.e., Lake Magadi, Lerai Forest (12.95 km^2^) and the Mandusi (7.77 km^2^) and Gorigor swamps (23.31 km^2^) and dense vegetation adjacent to the crater wall, notably along the northern and eastern edges of the crater. The unsurveyed Lerai Forest, Mandusi and Gorigor swamps collectively constitute about 16.7% of the crater floor. The same blocks have been used since the 1960s for the attempted total ground counts. The ground counts were done by one team per block composed of one driver, one observer and one recorder in a four-wheel drive vehicle driving along north-south oriented line transects that are approximately one kilometer apart. Hence there could have been animal movements while the counting teams travelled from one transect to the next. However, some movements should have been detectable and accounted for by the counters.

From 1981 to 1985 there were no counts. In 1986, the vehicle ground counts were resumed. Since 1987 each of the six teams has been supplied with a 1:50,000 map marked with the transects, a compass, binoculars and a mechanical counter. Each block takes six to eight hours to complete and all blocks are censused simultaneously to minimize chances of duplicate counts due to animal movements [22, 30, 35, 65–69]. Unpublished records of NCAA and NEMP for 1963–2012 provide most of the seasonal data on animal numbers. Count totals from all the six blocks were summed to obtain the count total for each species in each season in the crater. Distance sampling methods were not used and so potential variability in species detectability and its impact on the count totals was not quantified. However, given the large size of the target species and the facts that most of them prefer open grasslands and that large parts of the crater floor are open grasslands thus making animals highly visible, it is highly unlikely that the counts missed any major groups [37]. Consequently, even though confidence intervals cannot be estimated for total counts, the large proportion of the total populations counted should make the total counts sufficiently accurate to reliably detect trends. Even so, a few species that often occur in woodlands or swamps, particularly Black rhino, hippo (*Hippopotamus amphibius*), buffalo and waterbuck, could have been underestimated somewhat [22]. But if the bias in the population estimates remains fairly constant over time, especially because the same counting procedure was used, then it should have little impact on temporal trends.

Total photographic aerial counts were conducted in 1964, 1965, 1966, 1977, 1978 and 1988 [30].

In 1964 large herbivores were counted in the crater using attempted aerial total count. A total of 4h and 24 mins were flown during 18–19 February 1964. The counting team consisted of the pilot and one observer. The Crater floor was partitioned into 10 counting blocks using distinctive physical landmarks. Each block was counted using closely spaced, parallel flight lines at an average height of 305 m. But the two blocks encompassing the Lerai Forest and the Swamp were counted at a height of about 46 m and using even more closely spaced flight lines to spot Black rhino and elephants in the forest and hippos in the swamp. All blocks were counted on the first day of the count and adjacent blocks were counted successively to minimize the influence on the accuracy of the count of animal movements between blocks. Most of the animals were counted by eye but large or very large groups of wildebeest were counted from overlapping oblique aerial photographs. Thomson’s and Grant’s gazelle were lumped together as gazelles. One of the 10 blocks was recounted on the second day to check the record of the previous day [24]. The 1977 aerial count was conducted in the dry season on 20^th^ October 1977 and the 1978 count in the wet season on 12^th^ February 1978. Both total aerial counts were based on 10 km systematic sampling. Ground counts were conducted almost simultaneously with each of the two aerial total counts and demonstrated reasonable agreement between population size estimates for most species from both methods (S2 Data).

A systematic reconnaissance flight (SRF) or aerial strip-sample count was done in January-February 1980, which included warthogs for the first time [70]. During this regional survey, an intensive 10% sample count was carried out on the Ngorongoro Crater floor [70]. The SRF method involved flying transects at a consistent minimum height of 61 m above the ground level and ground speed of 128.8 km /hr compatible with safety, with observers recording the number of individuals of each animal species seen within 100 m strips on both sides of the aircraft. Groups of 10 or more animals were photographed and later counted after projecting the photos on to large screens. The visual estimates were corrected using counts from the photographs. Jolly’s method 2 for transects of unequal lengths [71,72] was used to estimate the population size for each species and its standard error.

Ecosystems Ltd [70] compared the population size estimates from the February 1980 SRF survey with estimates from the 1977/78 ground and aerial total counts. For wildebeest and zebra, the 1980 SRF estimates were comparable to, but slightly higher than, those for the 1977/78 ground and aerial total counts [70]. For wildebeest, this was attributed to calves not being counted in 1977/78. The population estimates for buffalo, eland and hartebeest were also comparable between the counts [70]. But the 1980 SRF Black rhino estimate was much higher than the ground counts but the 95% confidence limits were wide and the lower confidence limit overlapped with the estimates for the 1977/78 ground and aerial total counts. Lastly, the 1980 SRF estimate for elephant was far in excess of the ground estimates but the confidence limits for the SRF estimate were wide, complicating the comparison [70].

The ground counts for the 12 most common large herbivore species for the Ngorongoro Crater during 1963–2012 are provided in S2 Data. The same data set with the missing counts imputed using a state-space model is provided in S3 Data.

There are multiple potential problems that can undermine the reliability of attempted total counts. These include the likelihood of duplicate counting or under counting, lack of a measure of variance in population size estimates and likely violation of the assumption that all animals are detected and counted. Moreover, the assumption of perfect and constant detectability in space and time inherent in total counts was also likely violated. The attempted total counts or censuses have imperfect detectability in practice but variation in detectability was not quantified. Thus, although counters did their best to minimize double counting groups of animals, by systematically traversing counting blocks transect after transect, there were almost certainly some human errors, as with most animal counting methods. During 26–27 July 1986, ground total counts were carried out simultaneously with ground strip sample counts for some species using 100 m strips either side of the vehicles to obtain confidence limits for population size estimates. However, sample counting proved to be either impractical or impossible in practice over certain areas and produced excessively wide confidence limits for some species [30]. Ground total counts were replaced with distance sampling from vehicles in 2013 but results of these counts were unavailable.

Despite not accounting for imperfect detectability and not providing estimates of population variance, attempted total counts have been widely used for counting wildlife in African savannas since at least the 1950s. They continue to be used for continuity with historic counts and to estimate population sizes for rare species of conservation concern, or abundant and highly gregarious species, both of which are hard to reliably count using contemporary distance sampling methods. For example, attempted ground total counts have been used in Kenya to count animals every two months in Nairobi National Park since June 1960, in Lake Nakuru National Park since 1970 and in Nakuru Wildlife Conservancy since 1996 [73–75]. Attempted aerial total counts have also been used to count animals annually in the Kruger National Park in South Africa from 1977 to 1997 [76], in Masai Mara National Reserve of Kenya since 1962 and the Serengeti National Park since 1958 [12,77].

Attempted total counts can estimate population size for rare or abundant and gregarious animal species that are hard to count accurately using distance sampling for two reasons. First, distance sampling requires sightings of at least 60–80 groups of each animal species to accurately model detection functions, which is unachievable for most rare species. Thus, after attempted aerial total counts were substituted with aerial distance sampling in the Kruger National Park in 1998, it became difficult to obtain reliable estimates of population size for many species, including the warthog, wildebeest and waterbuck, even with a coverage intensity exceeding 22% of the park area [78]. Similarly, in the Nakuru Wildlife Conservancy of Kenya, distance sampling yielded insufficient sightings to model detection functions for many rare species. It was possible to estimate population size for some species only by pooling together several species of “similar size” and that “provide similar visual cues”. No population size estimates could be generated for species with too few sightings to model detection functions and that are hard to group with others [75]. The interpretation of such pooled estimates and their use in wildlife conservation and management can be contentious. Second, for abundant and highly gregarious species, such as wildebeest, zebra and Thomson’s gazelles in Serengeti-Mara that form groups spread over many kilometers, it is impractical to reliably estimate population size using distance sampling.

Total biomass for the wet and dry seasons for each year were calculated using unit weights in Coe et al. [7]. Biomass was calculated separately for each species and season. The fact that Black rhinos, elephants and warthogs move into the forest at the edge of the crater and into Lerai Forest make them more difficult to count and may affect their contribution to biomass.

### Ethics statement

All the animal counts in the Ngorongoro Crater were carried out as part of a long-term monitoring Program under the auspices of the Ngorongoro Conservation Area Authority (NCAA).

## Statistical modeling and analysis

### Modeling temporal variation in rainfall

For analysis, all counts conducted in each season (dry or wet) were assigned to the season, used to model trends for the season, and separately related to rainfall. The time series of rainfall was analyzed by using the unobserved components model (UCM), which is a special case of the linear Gaussian state space or structural time series model, to decompose the annual, wet season and dry season rainfall time series (r_t_) into their trend (μ_t_), cyclical (φ_t_), seasonal (δ_t_) and irregular (ϵ_t_) components

$$r_{t}=\mu_{t}+\varphi_{t}+\delta_{t}+\partial_{t}+\sum_{i=1}^{p}\theta_{i}r_{t-i}+\sum_{j=1}^{m}\beta_{j}x_{jt}+\epsilon_{t};\;t=1,2,\ldots ,n$$ (1)

in which ∂_t_ is the autoregressive component, $\sum_{i=1}^{p}\theta_{i}r_{t-i}$ is the autoregressive regression terms, β_j_ are the explanatory regression coefficients, x_jt_ are regression variables treated as fixed effects and (*ϵ*_*t*_) are independent and identically (*i*.*i*.*d*.) normally distributed errors or disturbances having zero mean and variance $\sigma_{\epsilon }^{2}$. This is equivalent to assuming that ϵ_t_ is a Gaussian white noise process. The different model components are assumed to be statistically independent of each other.

We first assume a random walk (RW) model for the time trend, or equivalently that the trend ($\mu_{t}$) remains approximately constant through time. The RW trend model can be specified as

$$\mu_{t}=\mu_{t‐1}+\eta_{t}$$ (2)

where $\eta_{t}∼i.i.d.N(0,\sigma_{\eta }^{2})$. Note that $\sigma_{\eta }^{2}=0$ implies that μ_t_ = a constant.

Additionally, we assume a stochastic cycle (*φ*_*t*_) with a fixed period (p>2), a damping factor (*ρ*) and a time-varying amplitude and phase given by

$$\left[\begin{array}{c} \varphi_{t}\\ \varphi_{t}^{*} \end{array}\right]=\rho \left[\begin{array}{cc} cos\omega & sin\omega \\ -sin\omega & cos\omega \end{array}\right]\left[\begin{array}{c} \varphi_{t-1}\\ \varphi_{t-1}^{*} \end{array}\right]+\left[\begin{array}{c} \upsilon_{t}\\ \upsilon_{t}^{*} \end{array}\right]$$ (3)

where 0<ρ≤1, ω = 2 × π/p, is the angular frequency of the cycle, υ_t_ and $\upsilon_{t}^{*}$ are independent Gaussian disturbances with zero mean and variance $\sigma_{\upsilon }^{2}$ and 0<ω<π. Values of ρ, p and $\sigma_{\upsilon }^{2}$ are estimated from the data alongside the other model parameters. The damping factor ρ governs the stationarity properties of the random sequence φ_t_ such that φ_t_ has a stationary distribution with mean zero and variance $\sigma_{\upsilon }^{2}/\left(1‐\rho^{2}\right)$ if ρ<1 but is nonstationary if ρ = 1. We specified and tested for significance of up to three cycles in the annual, wet season and dry season rainfall components.

Besides the random walk model (2), we modelled the trend component using a locally linear time trend incorporating the level and slope components and specified by

$$\mu_{t}=\mu_{t-1}+\beta_{t-1}+\eta_{t},\;\eta_{t}∼i.i.d.(0,\sigma_{\eta }^{2})$$ (4)

$$\beta_{t}=\beta_{t-1}+\xi_{t},\;\xi_{t}∼i.i.d.(0,\sigma_{\xi }^{2}),$$

where the disturbance variances $\sigma_{\eta }^{2}$ and $\sigma_{\xi }^{2}$ are assumed to be independent. The UCM models (1) and (4), without the seasonal and regression components, were fitted by the diffuse Kalman filtering and smoothing algorithm [79] in the SAS UCM procedure [80].

We grouped years with the annual rainfall falling within the 0–10, 11–25, 26–40, 41–75, 76–90, 91–95 and 96–100^th^ percentiles of the frequency distribution of the annual rainfall as extreme, severe or moderate drought years, normal, wet, very wet or extremely wet years, respectively. The dry (June to October) and wet (November to May) seasons were similarly grouped [19]. These percentiles allowed us to quantify the degree of rainfall deficit or surfeit and represent the expected broad transitions in rainfall influences on vegetation production and quality in each year and season.

### Modeling trends in animal population size and biomass

Time trends in count totals for the 12 most common large herbivore species were modeled simultaneously using a multivariate semiparametric generalized linear mixed model assuming a negative binomial error distribution and a log-link function [50,53]. The variance of the negative binomial distribution model var(*y*) was specified as a quadratic function of the mean (μ), var(y) = μ(1+μ/k), where k is the scale parameter. The semi-parametric model is highly flexible and able to accommodate irregularly spaced, non-normal and overdispersed count data with many zeroes or missing values. The parametric part of the model contains only the main effect of animal species to allow direct estimation of the average population sizes for the different species in each season. The non-parametric part of the model contains two continuous random effects, each of which specifies a penalized spline variance-covariance structure. The first random spline effect fits a penalized cubic B-spline (P-spline, [81] with a third-order difference penalty to random spline coefficients common to all the 12 species and therefore models the temporal trend shared by all the species. The second random spline effect fits a penalized cubic B-spline with random spline coefficients specific to each species and thus models the temporal trend unique to each species. Each random spline effect had 20 equally spaced interior knots placed on the running date of the surveys (1963,…,2012) plus three evenly spaced exterior knots placed at both the start date (1963) and end date (2012) of the surveys. De Boor [82] describes the precise computational and mathematical properties of B-splines. The specific smoothers we used derive from the automatic smoothers described in Ruppert, Wand and Carroll [83].

The full model contains three variance components to be estimated, corresponding to the random spline time trend common to all species, random spline effects for the time trend specific to each species and the scale parameter for the negative binomial distribution. The full trend model was fitted by the residual penalized quasi-likelihood (pseudo-likelihood) method [84] in the SAS GLIMMIX procedure [80]. More elaborate details on this approach to modelling animal population trends can be found in Ogutu et al. [72]. Separate trend models were fit to the wet and dry season count totals for simplicity. The denominator degrees of freedom for Wald-type F-tests were approximated using the method of Kenward and Roger [85]. Temporal trends in total biomass calculated using unit weights in Coe et al. [7] were similarly modeled, separately for each season.

We used constructed spline effects to estimate and contrast population sizes for each species between 1964 versus 1974 when the Maasai and their livestock were evicted from the crater and 1974 versus 2012. The constructed spline effects consisted of a cubic B-spline basis with three equally spaced interior knots. A constructed regression spline effect expands the original time series of animal survey dates into a larger number of new variables (seven in this specific case). Each of the new variables is a univariate spline transformation. The constructed spline effects are special model effects, in contrast to classical classification or continuous effects, and can be constructed using various other basis functions, including the truncated power function basis. These special model effects allowed estimation of the expected counts of each animal species at specified values of time (1964, 1974 and 2012). Because of the two comparisons made for each species, a multiplicity correction was made to control the familywise Type I error rate. We thus computed simulation-based step-down-adjusted *p*-values [86].

### Relating animal population size to rainfall

Population size was related to cumulative moving averages of the past annual, wet season and dry season rainfall components each computed over 1, 2,…,6 years for a total of six different moving averages per rainfall component. The maximum of 6-year window was chosen to match the approximately 5-year dominant periodicity or quasi-cyclical pattern estimated for the time series of the wet season and annual rainfall components (S3 Fig), based on the UCM model and spectral functions evaluated by the finite Fourier transform method. Spectral densities were obtained by smoothing the raw spectra or periodograms using moving average smoothing with weights derived from the Parzen kernel [80].

The moving rainfall averages index changing habitat suitability for ungulates associated with carry-over effects of prior rainfall on vegetation conditions. Population sizes were related to each of the 18 moving averages using a generalized linear model assuming a negative binomial error distribution and a log link function. The following six different functional forms were used for each of the 18 moving averages [11]:

μ=exp(αr) (5)

$$\mu =exp(\alpha r+\beta r^{2})$$ (6)

μ=exp(αln(r)) (7)

μ=exp(αr+βln(r)) (8)

$$\mu =exp(\alpha r+\beta r^{2}+\gamma ln(r))$$ (9)

μ=exp(αr+βln(r)+γrln(r)) (10)

These models were selected to represent (1) a linear increase or decrease in animal population size with increasing rainfall, (2) an increase in animal abundance with increasing rainfall up to some asymptote, or (3) an increase in animal abundance with increasing rainfall up to a peak at some intermediate levels of rainfall, followed by decline with further increase in rainfall [11]. The most strongly supported rainfall component, specific moving average and functional form were then selected using the corrected Akaike Information Criterion (AICc, [87], S13 and S14 Tables).

### Forecasting animal population dynamics using projected future climate

The relationships established between the time series of historic animal counts in the wet and dry seasons and lagged wet and dry season rainfall series were used to forecast the likely future trajectories of the wet and dry season population size for each species under three alternative climate change scenarios. We used the (Vector Autoregressive Moving Average Processes with exogeneous variables) VARMAX model to model the dynamic relationships between the wet and dry season counts of each species and the lagged wet and dry season rainfall and to forecast the seasonal animal counts. The model is very general and highly flexible and allows for the following among other features. 1) Modelling several time series of animal counts simultaneously. 2) Accounting for relationships among the individual animal count component series with current and past values of the other series. 3) Feedback and cross-correlated explanatory series. 4) Cointegration of the component animal series to achieve stationarity. 5) Seasonality in the animal count series. 6) Autoregressive errors. 7) Moving average errors. 8) Mixed autoregressive and moving average errors. 9) Distributed lags in the explanatory variable series. 10). Unequal or heteroscedastic covariances for the residuals.

The VARMAX model incorporating an autoregressive process of order p, moving average process of order q and in which the number of lags of exogenous (independent) predictor variables s is denoted as VARMAX(p,q,s). Since some animals move seasonally between the Ngorongoro Crater and the surrounding multiple use areas, the wet and dry season counts do not estimate the same underlying population size. We therefore treat the wet and dry season counts as two separate but possibly correlated variables and use a bivariate VARMAX(p,q,s) model. We allow variation in herbivore numbers in the wet and dry season to depend on the total wet and dry season rainfall in the current year (t) and in the preceding five years (*t*-1,…, *t*-5). The model thus allows the current wet and dry season rainfall components and their lagged values up to five years prior to the current count year to influence the population size of herbivores in the current wet and dry season. The model can also therefore be viewed as a multiple (or distributed) lag regression model. The VARMAX (p,q,s), model we used to forecast the future population dynamics of the five most abundant herbivore species can thus be cast as:

$$N_{t}=\sum_{j=1}^{p}\Phi_{j}N_{t-j}+\sum_{j=0}^{s}\Omega_{j}^{}x_{t-j}+\epsilon_{t}-\sum_{j=1}^{q}\Omega_{j}\epsilon_{t-j}$$ (11)

where N_t_ = (N_wet,t_, N_dry,t_)^T^ are the population sizes of the same species in the wet and dry seasons at time *t*, ***x***_*t*_ = (*wet*_*t*−0_,…,*wet*_*t*−5_,*dry*_*t*−0_,…,*dry*_*t*−5_)^*T*^ are the wet and dry season rainfall components divided by their long-term means and lagged over 0 to 5 years. ϵ_t_ = (ϵ_wet,t_, ϵ_dry,t_)^T^ are a two-dimensional vector white noise process. It is assumed that $E(\epsilon_{t})=0,\;E(\epsilon_{t}\epsilon_{t}^{T})=\Sigma$ and $E(\epsilon_{t}\epsilon_{u}^{T})$ for t≠u. We further assume that p and q are each equal to either 1 or 2 whereas s is set equal to 5. Accordingly, the model can be denoted symbolically as a VARMAX (2,2,5) model. In other words, in order to project the population dynamics of the Ngorongoro large herbivores, we built a model relating the population size of each herbivore species in the current year (t) to the population size in the past one to two years (year *t*-1 and *t*-2; i.e., autoregressive process of order p = 1 or 2). The model also allows residuals for the current year to depend on the residuals for the previous one to two years (i.e. a moving average process of order q = 1 or 2). Since herbivore numbers are counted once in the wet season and once in the dry season of each year we did not allow for seasonal variation in the counts.

The VARMAX (p,q,s) model can be represented in various forms, including in state space and dynamic simultaneous equation or dynamic structural equations forms. We used bivariate autoregressive moving average models with the wet and dry season rainfall as the explanatory variables. We tested and allowed for various lags in rainfall so that the models can be characterised as autoregressive and moving-average regression with distributed lags. We also used dead-start models that do not allow for present (current) values of the explanatory variables. We tested for heteroscedasticity in residuals and tested the appropriateness of GARCH-type (generalized autoregressive conditional heteroscedasticity) conditional heteroscedasticity of residuals. We used several information-theoretic model selection criteria as aids to determine the autoregressive (AR) and moving average (MA) orders of the models. The specific criteria we used were the Akaike information criterion (AIC), the corrected AIC (AICc) and the final prediction error (FPE). As additional AR order identification aids, we used partial cross-correlations for the response variable, Yule-Walker estimates, partial autoregressive coefficients and partial canonical correlations. Parameters of the selected full models were estimated using the maximum likelihood (ML) method. Roots of the characteristic functions for both the AR and MA parts (eigenvalues) were evaluated for their proximity to the unit circle to infer evidence for stationarity of the AR process and inevitability of the MA process in the response series [80].

The adequacy of the selected models was assessed using various diagnostic tools. The specific diagnostic tools we used are the following. 1) Durbin-Watson (DW) test for first-order autocorrelation in the residuals. 2) Jarque-Bera normality test for determining whether the model residuals represent a white noise process by testing the null hypothesis that the residuals are normally distributed. 3) F tests for autoregressive conditional heteroscedastic (ARCH) disturbances in the residuals. This F statistic tests the null hypothesis that the residuals have equal covariances. 4) F tests for AR disturbance computed from the residuals of the univariate AR(1), AR(1,2), AR(1,2,3) and AR(1,2,3,4) models to test the null hypothesis that the residuals are uncorrelated. 5) Portmanteau test for cross correlations of residuals at various lags. Final forecasts and their 95% confidence intervals were then produced for the animal population size series for each of the five most common species in each season for lead times running from 2013 up to 2100.

In the table of the parameter estimates for the bivariate VARMAX (2,2,5) model fitted to the two time series of herbivore population size in the wet and dry seasons (S1 Table), the five lagged dry and wet season rainfall components (rightmost column labelled variable) for the current year (year t) up to five years prior to the current year (years t-1,…,t-5) are denoted by dry (t),…, dry (t-5) and wet (t),…, wet (t-5), respectively. Analogously, for the dry season counts, the autoregressive process of order 2 is denoted by, e.g., wildebeest_dry_(t-1) and wildebeest_dry (t -2) while the moving average process of order 2 by e1(t-1) and e2 (t-2). A parallel notation is used for the wet season counts. The estimated regression coefficients (estimate) for the parameters associated with each of these variables plus the intercept (Const1), the standard errors of the estimates and a *t*-test (*t*-value) of the null hypothesis that each coefficient is not significantly different from zero (Pr >|*t*|) are also provided in S1 Table. Furthermore, the estimated roots of the autoregressive (S2 Table) and moving average (S3 Table) processes are provided. It is important to note that the population of each herbivore species in the wet season of the current year depends not only on its lagged values in the preceding one to two years and on the current and past values of rainfall, but also on the population of the same herbivore species in the dry season lagged over the past one to two years. The same applies to the population of each herbivore species in the current dry season. This interdependence of the two series on each other is made possible because of the bivariate nature of the VARMAX (p,q,s) model. This model was fitted to the population counts of the herbivores for the wet and dry seasons for the period 1964–2012 based on historic station rainfall data for 1963 to 2012. Note that the historic total wet season rainfall component was divided by its mean for use in the model. The same was done for the total dry season rainfall component. Future forecasts were then produced by supplying the projected wet and dry season rainfall values, each divided by its mean, for Ngorongoro for 2013 to 2100.

Several univariate model diagnostics were used to extensively assess how well the selected bivariate VARMAX (p,q,s) model fitted the count data (S4–S7 Tables). The first model diagnostic tool, the Portmanteau Test for Cross Correlations of Residuals (S4 Table) was significant, considering only up to lag 5 residuals. This test of whether the residuals are white noise residuals (i.e. uncorrelated) based on the cross correlations of the residuals, suggests that the residuals were apparently correlated, when only up to lag 5 residuals are considered. Even so, results of the univariate model ANOVA diagnostics suggest that the models for both the dry and wet season counts were highly significant and had high predictive power (*r*^2^, S5 Table). Results of the Univariate Model White Noise Diagnostics (S6 Table) suggest that the residuals are normally distributed (Jarque-Bera normality test) and have equal covariances (ARCH (1) disturbances test). The Univariate AR Model Diagnostics indicate that the residuals are uncorrelated, contrary to the finding of the multivariate Portmanteau test (S7 Table). The modulus of the roots (eigenvalues) of the AR characteristic polynomial are less than 1 suggesting that the series are stationary. These tests suggest that the fitted models are reasonable. The log-transformed animal count totals, rainfall deviates, projected rainfall and forecast animal count totals (log scale) are provided in S4 Data. The SAS program codes used to analyze the rainfall data are provided in S1 Text while the code for analyzing the animal counts is provided in S2 Text.

## Results

### Rainfall

**Fig 2:**
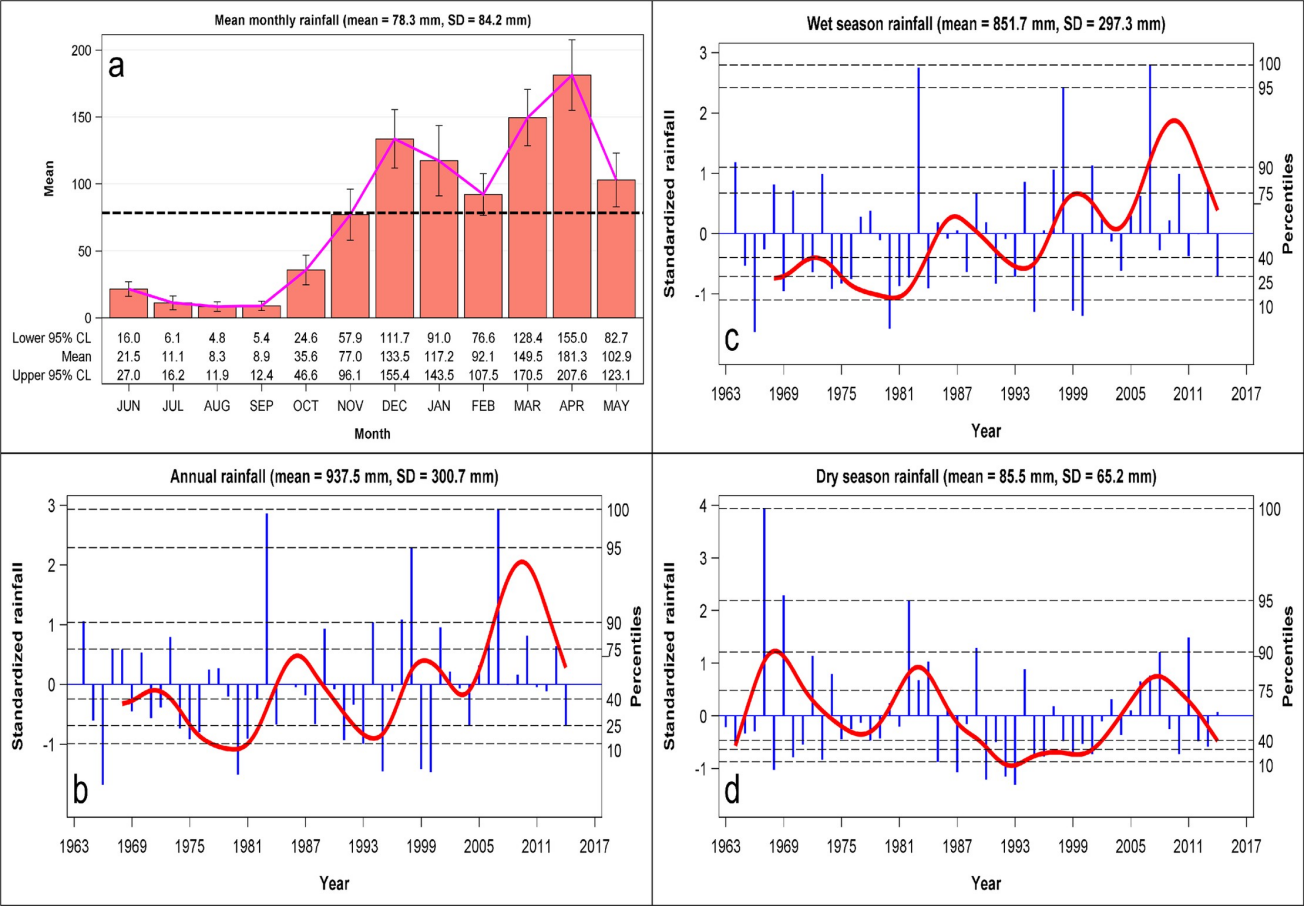
The distribution of a) total monthly rainfall (mean ± 1sd = 78.3 ± 84.2 mm) across months in the Ngorongoro Crater averaged over 1963–2014 and the interannaul variation in standardized deviations of the b) annual rainfall (mean ± 1SD = 937.5 ± 300.7 mm), c) wet season rainfall (mean± 1SD = 851.7 ± 297.3 mm), and d) dry season rainfall (mean± 1SD = 85.5 ± 65.2 mm) in the Ngorongoro Crater during 1963–2014. The vertical needles are the standardized deviates, the solid curves are the 5-year (annual and wet season) and 2-year (dry season) moving averages and the dashed horizontal lines are percentiles of the frequency distributions of the rainfall deviates.

Rainfall can be subdivided into the dry and wet season components. The dry season occurs from June to October whereas the wet season occurs from November to May. The wet season rainfall component is strongly bimodal, with the two modes corresponding to peaks in the long rains and the short rains. The major peak in rainfall occurs in April during the long rains (January-May) whereas the minor peak occurs in December during the short rains (November-December, Fig 2A). The total monthly rainfall averaged 78.3 ± 84.2 mm and was highly variable (%CV = 107.5%) during 1963–2014 (Fig 2A). The total annual rainfall averaged 937.5 ± 300.7 mm during 1963–2014 (Fig 2B) out of which the wet season rainfall (851.7 ± 297.3 mm) contributed 90.9% (Fig 2C) and the dry season rainfall (85.5 ± 65.2 mm) a mere 10.1% (Fig 2D). There were also considerable interannual variations in the annual, wet and dry season rainfall components (Fig 2B–2D). Smoothing of the time series of the total monthly rainfall exposed substantial variation with periods of below-average rainfall centered around 1966, 1975, 1980 and 1999 (S1 Fig).

Analysis of the annual rainfall showed that extreme droughts occurred in 1966, 1980, 1993, 1995, 1999 and 2000 while severe droughts were recorded in 1974–1976, 1981, 1991, 2004 and 2014. Further, the extremely wet years were 1983 and 2007 whereas very wet years were 1964, 1997 and 1998. Analysis of the wet season rainfall identified the same extreme and severe droughts and very wet years as the annual rainfall did (S8 Table, S2 Fig). In addition, the wet seasons of 1966, 1969, 1980, 1995, 1999 and 2000 experienced an extreme drought while the 1974,1975,1976, 1980,1981,1984, 1991 wet seasons had severe droughts. The dry seasons of 1968, 1985, 1987, 1990, 1992 and 1993 were extremely dry and the dry seasons of 1970, 1973, 1995, 1996, 1999, 2001 and 2010 were severe droughts. By contrast the dry seasons of 1967, 1969, 1982, 1989 and 2011 were either extremely wet or very wet (S8 Table, S2 Fig).

There were significant quasi-cyclic oscillations in the three rainfall components with approximate cycle periods of 4.64, 4.64 and 2.47 years for the annual, wet season and dry season rainfall components, respectively, based on spectral analysis (S9 Table, S3 Fig). Based on the unobserved components model (UCM), the dominant oscillation had a 4.83-year cycle for the annual rainfall, 3.82-year cycle for the wet season rainfall and 2.45-year cycle for the dry season rainfall (S10 Table, S4 Fig). In addition, there were secondary cycles in the wet and dry season rainfall components with approximate cycle periods of 2.2 years for the wet season component and 11.3 years for the dry season component (S10 Table, S4 Fig). The estimated damping factors for the cycles were all less than 1 except for the annual rainfall with a 4.83- year cycle and the wet season rainfall with a 2.2-year cycle both of which had damping factors equal to 1 (S10 Table, S4 Fig). The two cycles with damping factors equal to 1 are persistent whilst the remaining cycles with damping factors smaller than 1 are transient.

The disturbance variances for the irregular components for the wet and dry season rainfall, but not for the annual rainfall, were close to zero and statistically insignificant. This implies that the irregular components for the two seasonal rainfall components were deterministic whereas the irregular component for the annual rainfall was stochastic. Moreover, the estimated disturbance (error) variances for the cyclical components were significant for the 3.83-year cycle for the wet season and for both cycles for the dry season but not for the 4.83-year cycle for the annual rainfall (S10 Table, S4 Fig). These features jointly imply that the 4.83-year cycle identified for the annual rainfall is persistent and deterministic whereas the cycles identified for both the wet and dry season rainfall are stochastic and transient (S10 Table, S4 Fig). Even so, significance tests of the disturbance (error) variances of the cyclical components in the model at the end of the estimation span indicate that the disturbance variances for the cycle in the annual rainfall component and both cycles in the wet season rainfall component were significant but those for the two cycles in the dry season rainfall component were insignificant (S11 Table). Since the 4.83-cycle in the annual rainfall component is deterministic the additional significant test result means that the annual cycle is indeed significant. The significant disturbance variances for the two stochastic cycles in the wet season rainfall component (S11 Table) applies only to the part of the time series of wet season rainfall near the end of the estimation span.

The disturbance terms for the level component for all the three rainfall components were significant only for the wet season but not for the annual or dry season rainfall. As well, the slope component was significant only for the wet season rainfall (S11 Table, S4 Fig). This implies that, of the three rainfall components, only the wet season rainfall increased systematically over time in Ngorongoro (S11 Table, S4 Fig). The smoothed rainfall cycles in the three rainfall components further reinforce the conclusion that the oscillation in annual rainfall is persistent and deterministic whereas the oscillations in the wet and dry season rainfall are transient and stochastic (S4 Fig).

### Projected rainfall and temperatures

The projected annual rainfall showed no evident systematic trend under all the three scenarios. However, the general average rainfall level is consistently and substantially higher under the RCP 2.6 than the RCP 4.5 and 8.5 scenarios. The RCP 4.5 and 8.5 scenarios have comparable average levels but RCP 4.5 is expected to receive somewhat more rainfall. Notably, rainfall shows marked inter-annual variation characterized by sustained quasi-cyclic oscillations during 2006–2100 regardless of scenario (S5 Fig).

The minimum and maximum temperatures are expected to rise during 2006–2100, on average, by 1, 2 and 6°C under the RCP 2.6, 4.5 and 8.5 scenarios, respectively. Consequently, the average maximum temperature is expected to increase during 2006–2100 from 23 to 24°C under RCP 2.6, 24 to 26°C under RCP 4.5 and 23 to 29°C under RCP 8.5. The average minimum temperature is similarly anticipated to rise during 2006–2100 from 14 to 15°C under RCP 2.6, 14 to 16°C under RCP 4.5 and 14 to 20°C under RCP 8.5 (S5 Fig).

### Historic herbivore population dynamics

**Fig 3:**
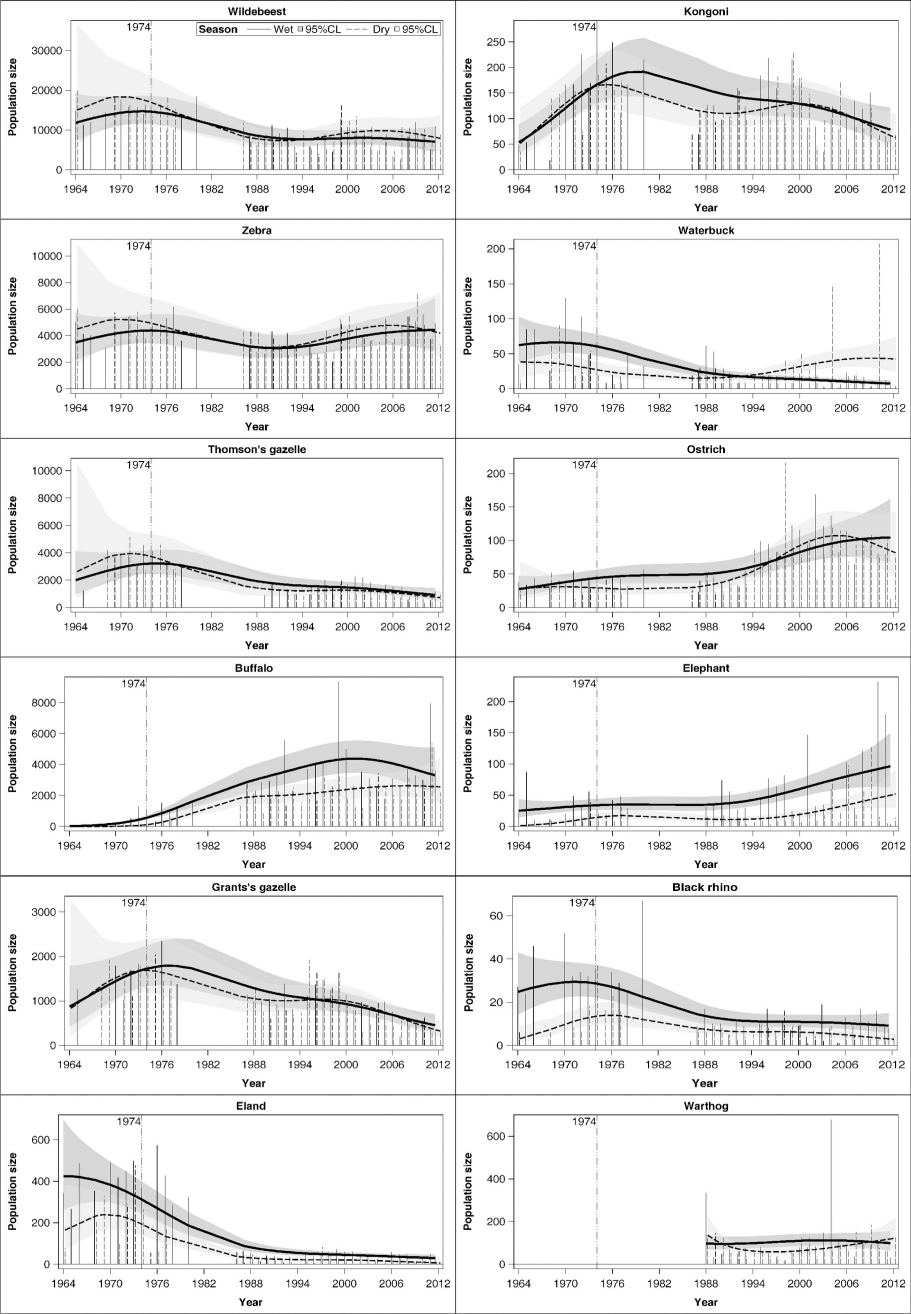
Trends in the population sizes of the 12 most common large herbivore species in the Ngorongoro Crater in the wet and dry seasons from 1964 to 2012. The vertical needles denote wet season (solid) and dry season (dashed) count totals. Thick solid and dashed curves denote the fitted wet season and dry season trend curves. The shaded regions are the 95% point wise confidence bands.

The population size of wildebeest, zebra, Thomson’s gazelle, Grant’s gazelle, kongoni (Coke’s hartebeest), and Black rhino increased from 1964 to a peak around 1974–1976 and then declined thereafter in both the wet and dry seasons. Eland and waterbuck had a general downward trend from the early 1970's. Zebra numbers increased again from 1995 to 2012 whereas Grant’s gazelle and kongoni numbers in the dry season increased again from 1995 to 2000 before declining further (Fig 3). In stark contrast to the other species, numbers of buffalo increased markedly following the removal of Maasai livestock from the crater in 1974. Elephant and ostrich numbers similarly increased in the crater, with substantial increase apparent in ostrich numbers following the extreme 1993 dry season drought (Fig 3). Buffalo, eland, elephant and Black rhino were more abundant in the crater in the wet than the dry season. There were far more eland and Black rhino in the crater in the wet season compared to the dry season in the 1970s than in the 2000s. Conversely, there were far more buffalo and elephants in the crater in the wet season compared to the dry season in the 2000s than there were in the 1970s (Fig 3). Zebra were the only species to have maintained similar population sizes from 1964 to 2012.

Comparisons of the expected population sizes between 1964 and 1974 as well as between 1974 and 2012 based on constructed spline effects showed that while some species increased significantly over time, others did not, or even declined. Species that increased slightly between 1964 and 1974 in the wet season were wildebeest, Grant’s gazelle, waterbuck and ostrich (S12 Table, Fig 3). Only buffalo, Thomson’s gazelle and kongoni numbers increased significantly between 1964 and 1974 in the wet season. Species that decreased slightly in numbers between 1964 and 1974 in the wet season were zebra, eland, elephant and Black rhino. Between 1974 and 2012, the numbers of Thomson’s gazelle, Grant’s gazelle, Black rhino, eland, kongoni and waterbuck decreased significantly in the wet season. In the same season and period, buffalo and elephant numbers increased. Zebra, wildebeest, and ostrich did not change noticeably (S12 Table, Fig 3). In the dry season, by contrast, numbers of some species either increased between 1964 and 1974 (buffalo, elephant, eland, kongoni), increased slightly (waterbuck) or tended to decrease (wildebeest, zebra, Thomson’s gazelle, Grant’s gazelle, ostrich). However, between 1974 and 2012 in the dry season, numbers of some species either increased (buffalo, ostrich), tended to increase (waterbuck), decreased (Thomson’s gazelle, Grant’s gazelle, Black rhino, eland, kongoni), or decreased slightly (wildebeest, zebra, elephant, S12 Table, Fig 3).

### Herbivore biomass dynamics

**Fig 4:**
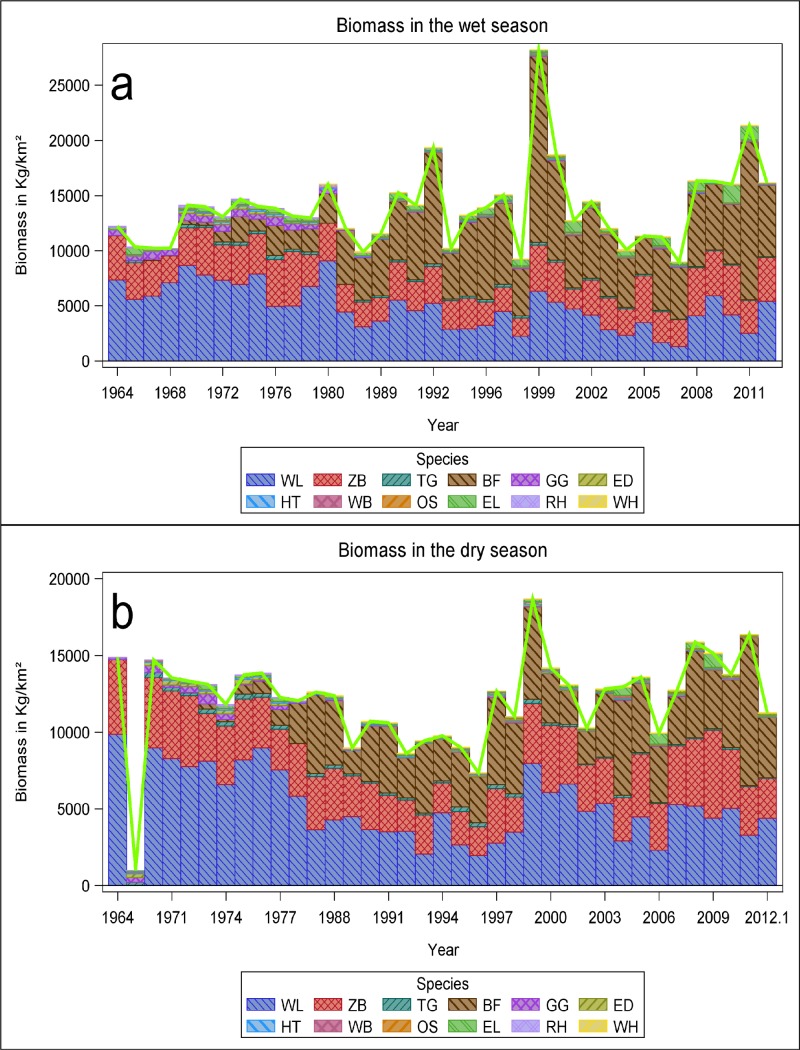
Temporal trends in the cumulative total biomass (kg) of the 12 most common large herbivore species in the Ngorongoro Crater during a) the wet season and b) the dry season during 1964 to 2012. The unit weights (in kg) are 1725, 816, 450, 340, 200, 160, 125, 123, 114, 45, 40 and 15 for elephant, Black rhino, buffalo, eland, zebra, waterbuck, kongoni, wildebeest, ostrich, warthog, Grant’s gazelle and Thomson’s gazelle, respectively. Note that wildebeest and zebra were not counted in the dry season of 1968. In years when multiple surveys were done in the same season (e.g., the wet season of 1966 or 1970), only the survey with the maximum count was used to calculate biomass.

Herbivore biomass in the wet season was initially dominated by wildebeest, followed by zebra. Following the eviction of the Maasai and their livestock from the crater in 1974, buffalo biomass increased relative to wildebeest and zebra to a peak during 1999–2000. After the 1999–2000 drought, the biomass of buffalo and the other herbivore species declined to the pre-drought levels. Nevertheless, wildebeest still makes a smaller contribution to the total biomass currently than they did before cattle left the crater and buffalo numbers were still low (Fig 4A). The relative increase of buffalo biomass compared to wildebeest and zebra was also apparent in the dry season biomass (Fig 4B).

The total herbivore biomass trends in the crater have been dynamic and relatively stable. During the dry season from 1964 to 1974 there was no significant change and this trend was also non-significant for the dry season from 1974 to 2011 (Table 1). This scenario of a non-significant trend from 1964 to 1974 and again from 1974 to 2011 was also consistent for the wet season (Table 1).

**Table 1:** The expected (predicted) aggregate large herbivore biomass in the wet and dry seasons of 1964, 1974 and 2011 and the difference between the 1964 vs 1974 and 1974 vs 2011 estimates and tests of significance of the differences based on constructed penalized cubic B-splines.

Statement	Label	Estimate	Standard Error	DF	t Value	Pr > |t|	Adjustment	Adj P
Number								
1	Dry Season at time = 1964	-63.3804	265.19	2.326	-0.24	0.8306		.
2	Dry Season at time = 1974	-41.5788	146.46	2.349	-0.28	0.7996		.
3	Dry Season at time = 2011	8.0933	0.1129	47.44	71.67	<0.0001		.
5	Wet Season at time = 1964	-64.2763	265.14	2.326	-0.24	0.8282		.
6	Wet Season at time = 1974	-41.5482	146.46	2.349	-0.28	0.7998		.
7	Wet Season at time = 2011	8.4434	0.1336	50.95	63.2	<0.0001		.
4	Diff for Dry Season at time = 1964 vs time = 1974	21.8017	123.24	2.309	0.18	0.8739	Simulated	0.9479
4	Diff for Dry Season at time = 1974 vs time = 2011	49.6721	146.46	2.349	0.34	0.7625	Simulated	0.8474
8	Diff for Wet Season at time = 1964 vs time = 1974	22.7281	123.16	2.309	0.18	0.8686	Simulated	0.9447
8	Diff for Wet Season at time = 1974 vs time = 2011	49.9916	146.46	2.348	0.34	0.761	Simulated	0.8463

### Relationship between herbivore population size and rainfall

**Fig 5:**
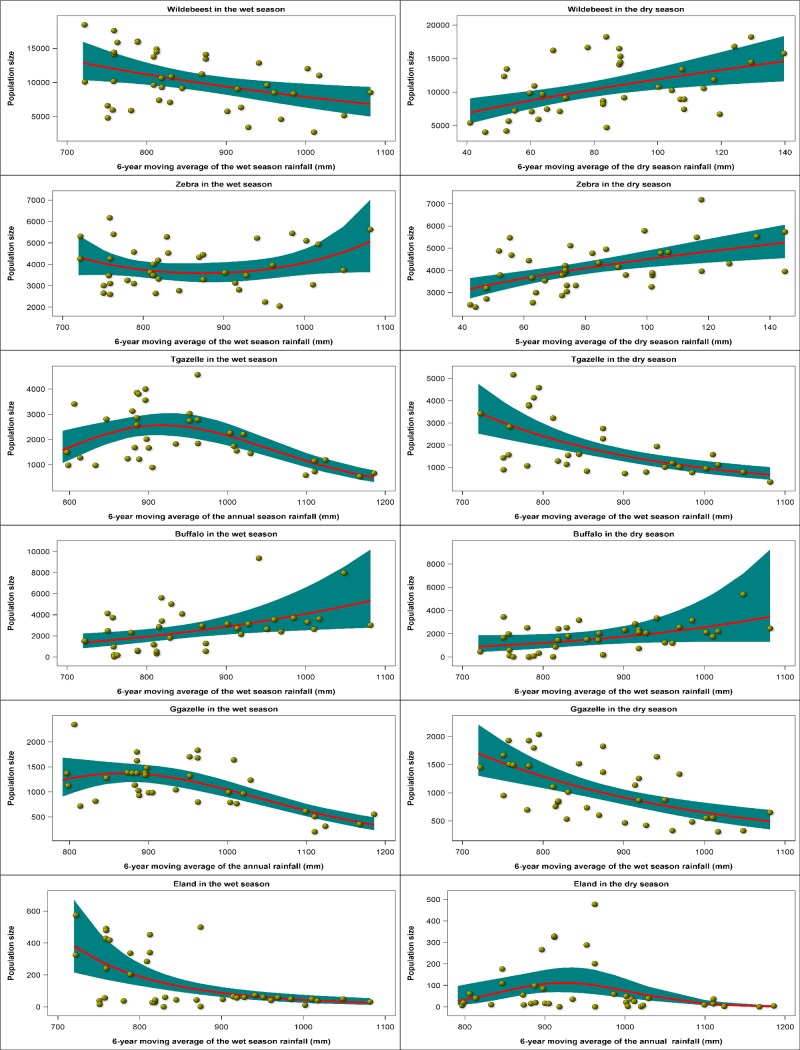
The corrected Akaike Information Criterion (AICc)-selected best regression relationships between the wet season and dry season count totals of wildebeest, zebra, Thomson’s gazelle, buffalo, Grant’s gazelle, and eland and the moving averages of the annual, wet season and dry season rainfall components for the Ngorongoro Crater during 1964–2012.

**Fig 6:**
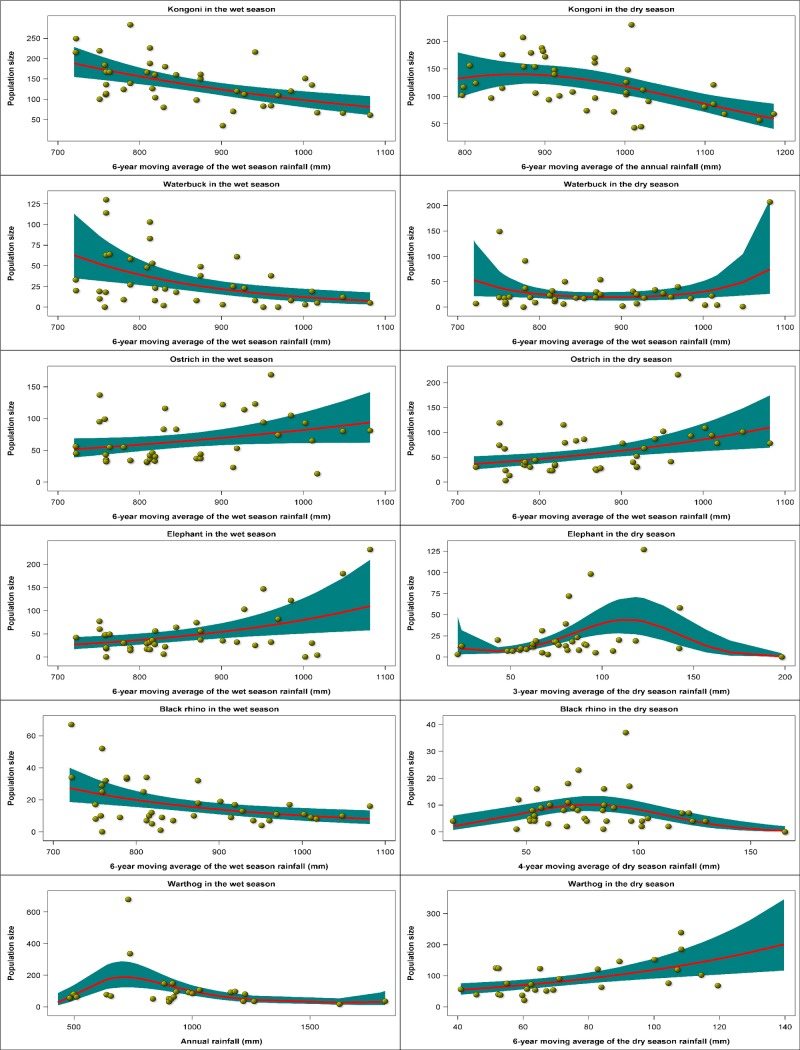
The corrected Akaike Information Criterion (AICc)-selected best regression relationships between the wet season and dry season count totals of kongoni, waterbuck, ostrich, elephant, Black rhino and warthog and the moving averages of the annual, wet season and dry season rainfall components for the Ngorongoro Crater during 1964–2012.

Herbivore population size was correlated with rainfall in both the wet and dry seasons. The particular rainfall component most strongly correlated with population size as well as the specific functional form of the relationship both varied with species and season (Figs 5 and 6, S13 and S14 Tables). In the wet season, population size was most tightly correlated with 1) 6-year moving averages of the wet season rainfall (wildebeest, zebra, buffalo, eland, kongoni, waterbuck, ostrich, elephant, Black rhino), 2) 6-year moving average of the annual rainfall (Thomson’s and Grant’s gazelles), or 3) the current annual rainfall (warthog). In the dry season, population size had the strongest correlation with 1) 6-year moving average of the wet season rainfall (Thomson’s and Grant’s gazelle, buffalo, waterbuck, ostrich), 2) 5-6-year moving average of the dry season rainfall (wildebeest, zebra, warthog), 3) 6-year moving average of the annual rainfall (eland, kongoni), or 4) 3-4-year moving average of dry season rainfall (elephant, Black rhino, Figs 5 and 6, S13 and S14 Tables). The dependence of population size on rainfall followed three general patterns. The first pattern is characterized by a decline in population size with increasing rainfall and is shown by wildebeest, eland, kongoni, waterbuck and Black rhino in the wet season, and Thomson’s gazelle, Grant’s gazelle and waterbuck in the dry season. The second pattern consists of an increase in population size with increasing rainfall and is shown by zebra, buffalo, ostrich and elephant in the wet season and wildebeest, zebra, buffalo, ostrich and warthog in the dry season. The third and last pattern is characterized by a humped relationship between population size and rainfall in which population size peaks at intermediate levels of rainfall and is shown by Thomson’s and Grant’s gazelles and warthog in the wet season and eland, kongoni, elephant and Black rhino in the dry season (Figs 5 and 6, S13 and S14 Tables).

### Projected herbivore population dynamics

**Fig 7:**
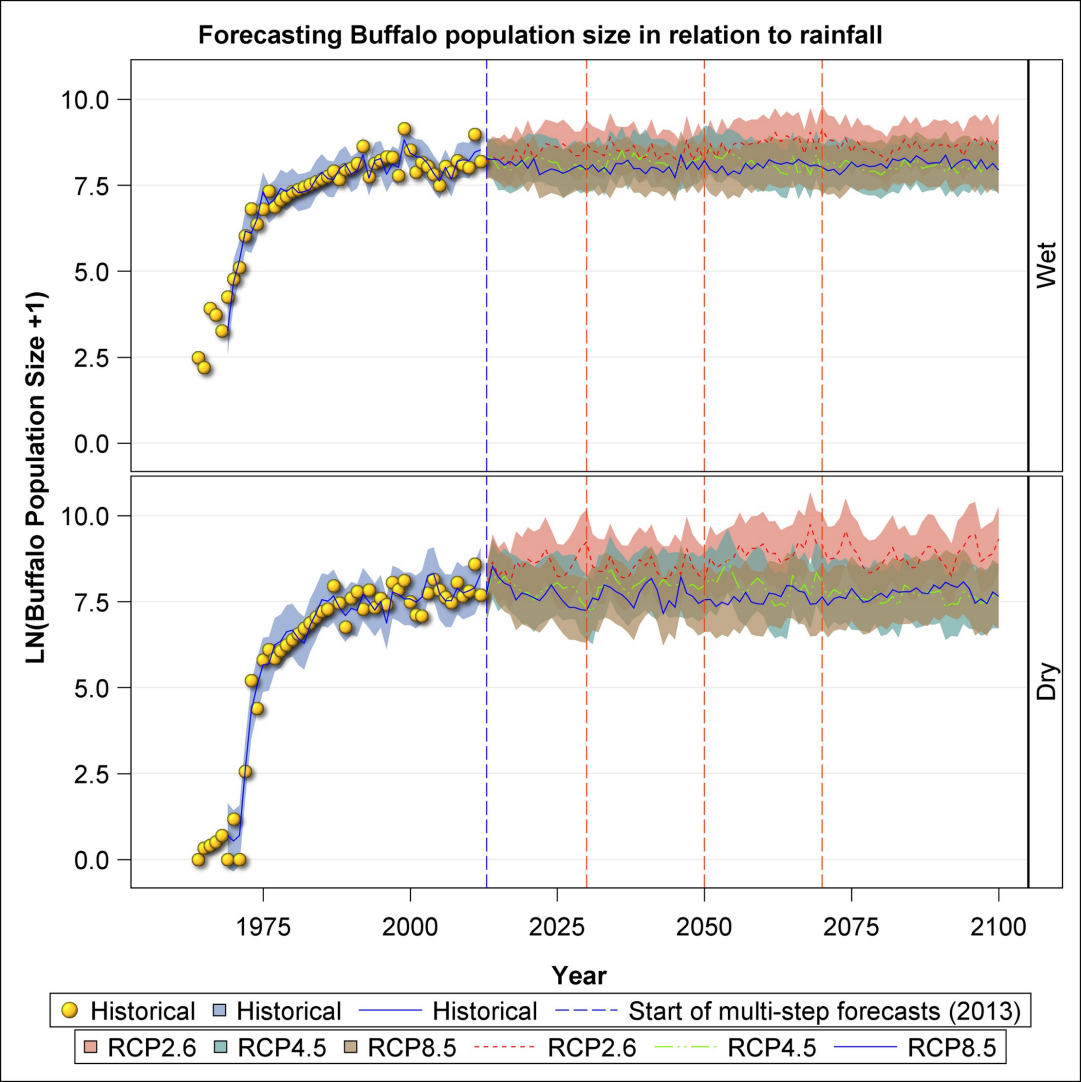
Historic and projected population size of buffalo in the Ngorongoro Crater during the wet and dry seasons based on the three climate change scenarios: RCP 2.6, RPC 4.5 and RCP 8.5.

The projected population trajectories suggest that under the RCP 2.6 scenario, buffalo numbers will likely continue to increase after 2012, albeit at a decelerating rate, towards 7000–11000 animals by 2100 (Fig 7). But the crater buffalo population is likely approaching its upper bound of about 4000 animals and will likely fluctuate about this number (4000) till 2100 under the RCP 4.5 and 8.5 scenarios regardless of season (Fig 7). As expected, the population of this large-sized bulk grazer is projected to be greatest on average under RCP 2.6, least under RCP 8.5 and intermediate under RCP 4.5 for both the wet and dry seasons (Fig 7).

**Fig 8:**
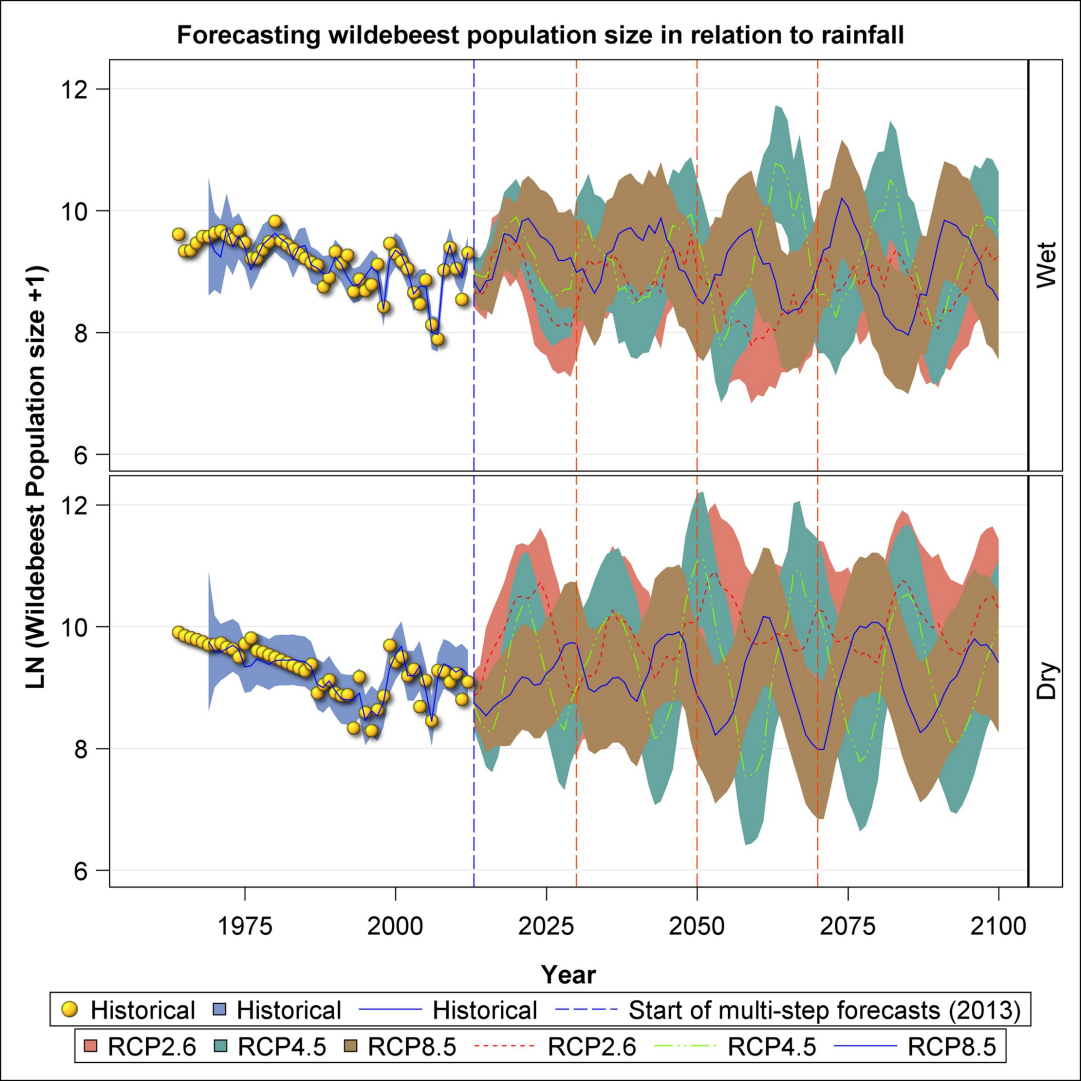
Historic and projected population size of wildebeest in the Ngorongoro Crater during the wet and dry seasons based on the three climate change scenarios: RCP 2.6, RCP 4.5 and RCP 8.5.

For wildebeest, the projected trajectories suggest strong and sustained oscillations in population size under all the three scenarios and both seasons, reflecting the strong projected rainfall oscillations (Fig 8). The oscillatory population dynamics in both the wet and dry seasons exhibited by wildebeest reveal extended periods of population increase followed by prolonged periods of persistent population declines. Nevertheless, there are also discernible differences in the projected population trajectories under the three climate change scenarios. The projected wildebeest population trajectories suggest that the population will continue to fluctuate widely between 5000 and 15000 animals in all the scenarios and seasons. It is only under the RCP 2.6 scenario that the dry season population shoots beyond 20000 animals around 2070 and 2090 (Fig 8). In the wet season, the projected average wildebeest abundance is greatest under RCP 4.5, intermediate under RCP 8.5 and lowest under RCP 2.6. In the dry season, however, wildebeest abundance is greatest on average under RCP 2.6, intermediate under RCP4.5 and lowest under RCP 8.5 (Fig 8).

**Fig 9:**
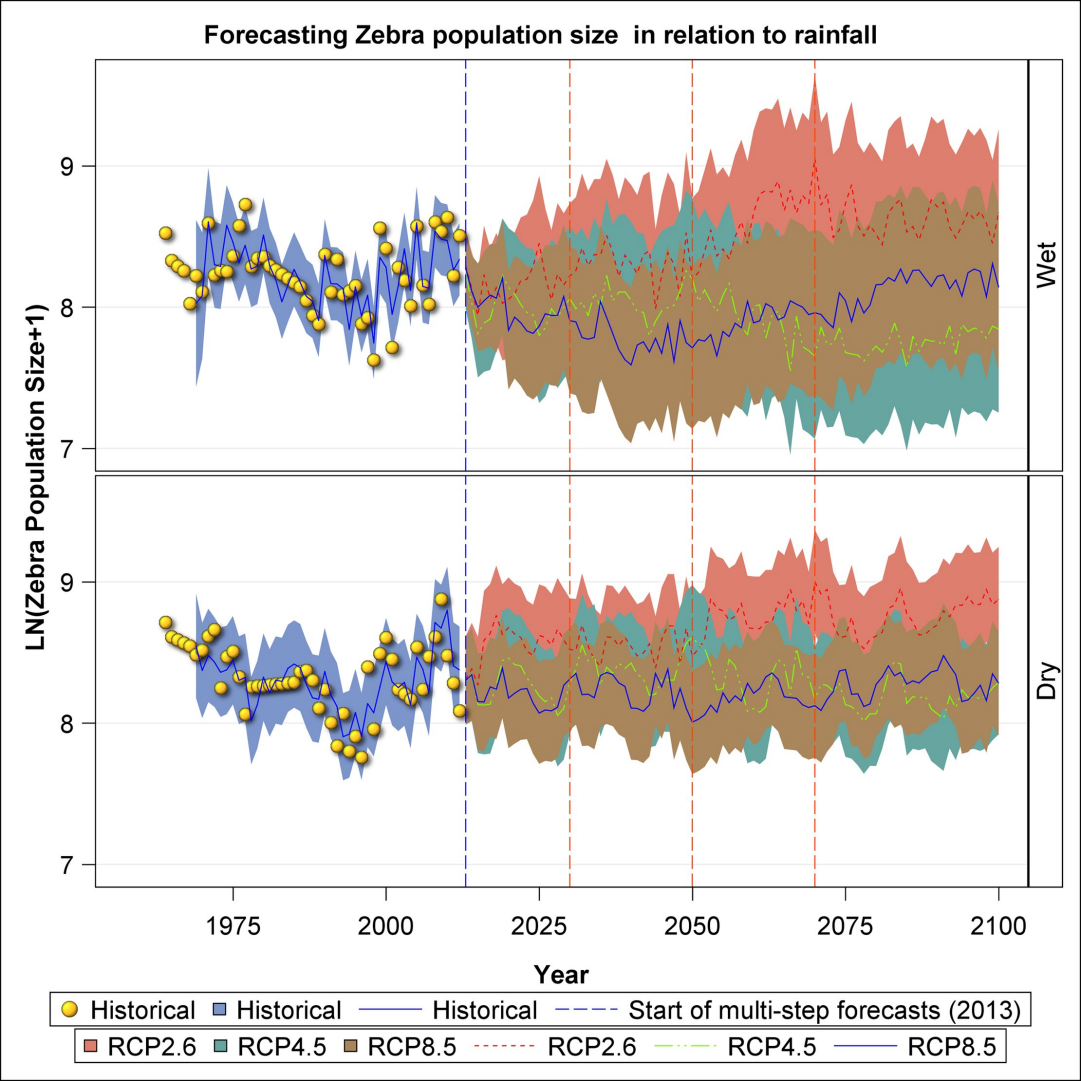
Historic and projected population size of Plains zebra in the Ngorongoro Crater during the wet and dry seasons based on the three climate change scenarios: RCP 2.6, RCP 4.5 and RCP 8.5.

The zebra population trajectories also reveal striking oscillations in population size under all the three scenarios, a general increase in population size under RCP 2.6 scenario in both seasons and a decrease and then increase in the RCP 8.5 scenario in the wet season (Fig 9). The zebra population size is projected to decline in the long term under the RCP 4.5 scenario in both seasons and the RCP 8.5 scenario in the dry season (Fig 9). In general, zebra will perform the best under RCP 2.6 and the worst under RCP 8.5. The performance of zebra under RCP 4.5 will be intermediate between RCP 2.6 and 8.5 from 2006 to around 2070 after which it will drop below that expected under RCP 8.5 (Fig 9).

**Fig 10:**
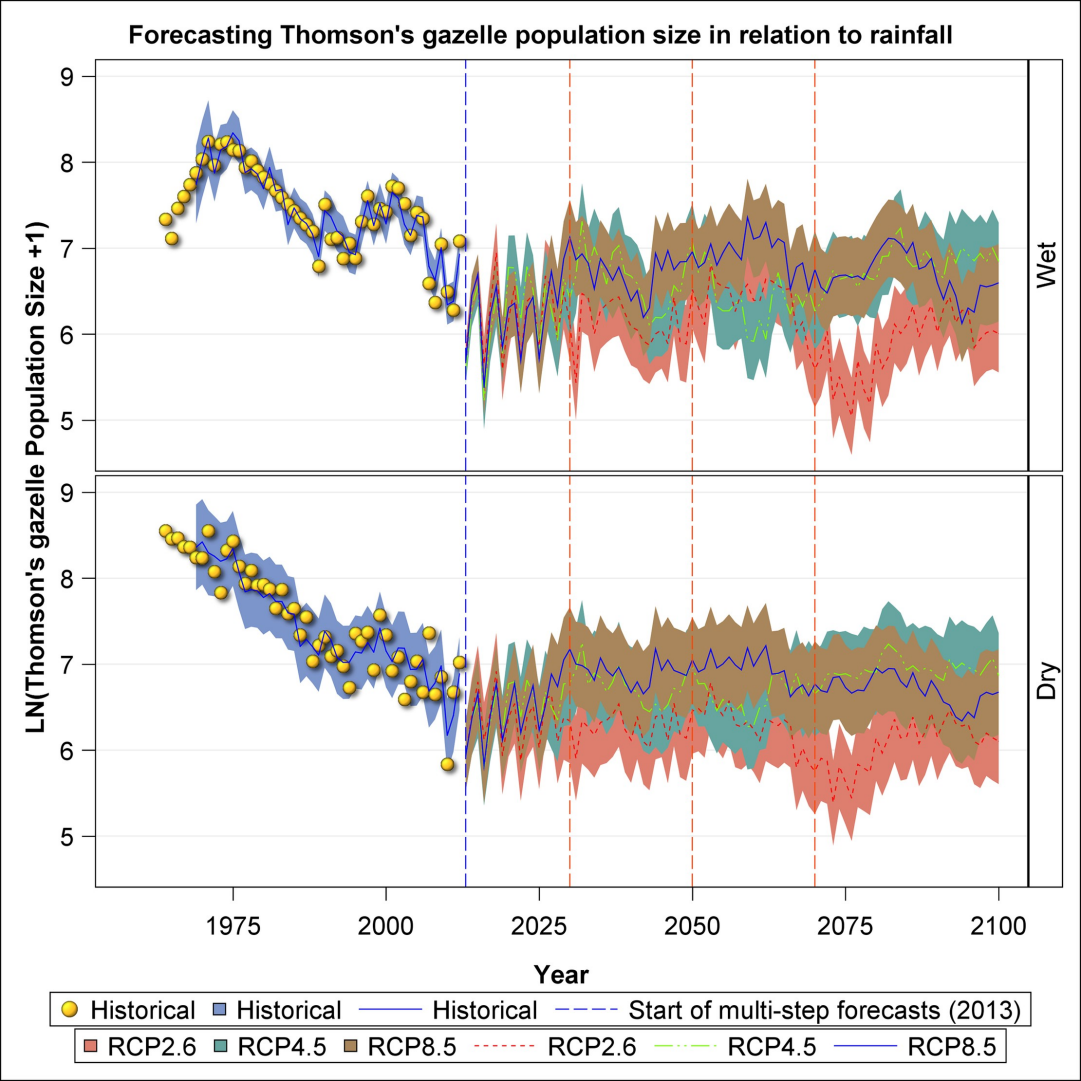
Historic and projected population size of Thomson’s gazelle in the Ngorongoro Crater during the wet and dry seasons based on the three climate change scenarios: RCP 2.6, RCP 4.5 and RCP 8.5.

The decline observed in historic Thomson’s gazelle numbers is projected to be persistent and to remain below the peak attained historically around 1974 under all scenarios and both seasons (Fig 10). Besides the general decline, Thomson’s gazelle numbers are projected to show persistent and marked oscillations irrespective of scenario or season. As predicted by their small body size and selective grazing, Thomson gazelles will likely perform the best under RCP 8.5 with the least rainfall, intermediately under RCP 4.5, and the worst under RCP 2.6 (Fig 10).

**Fig 11:**
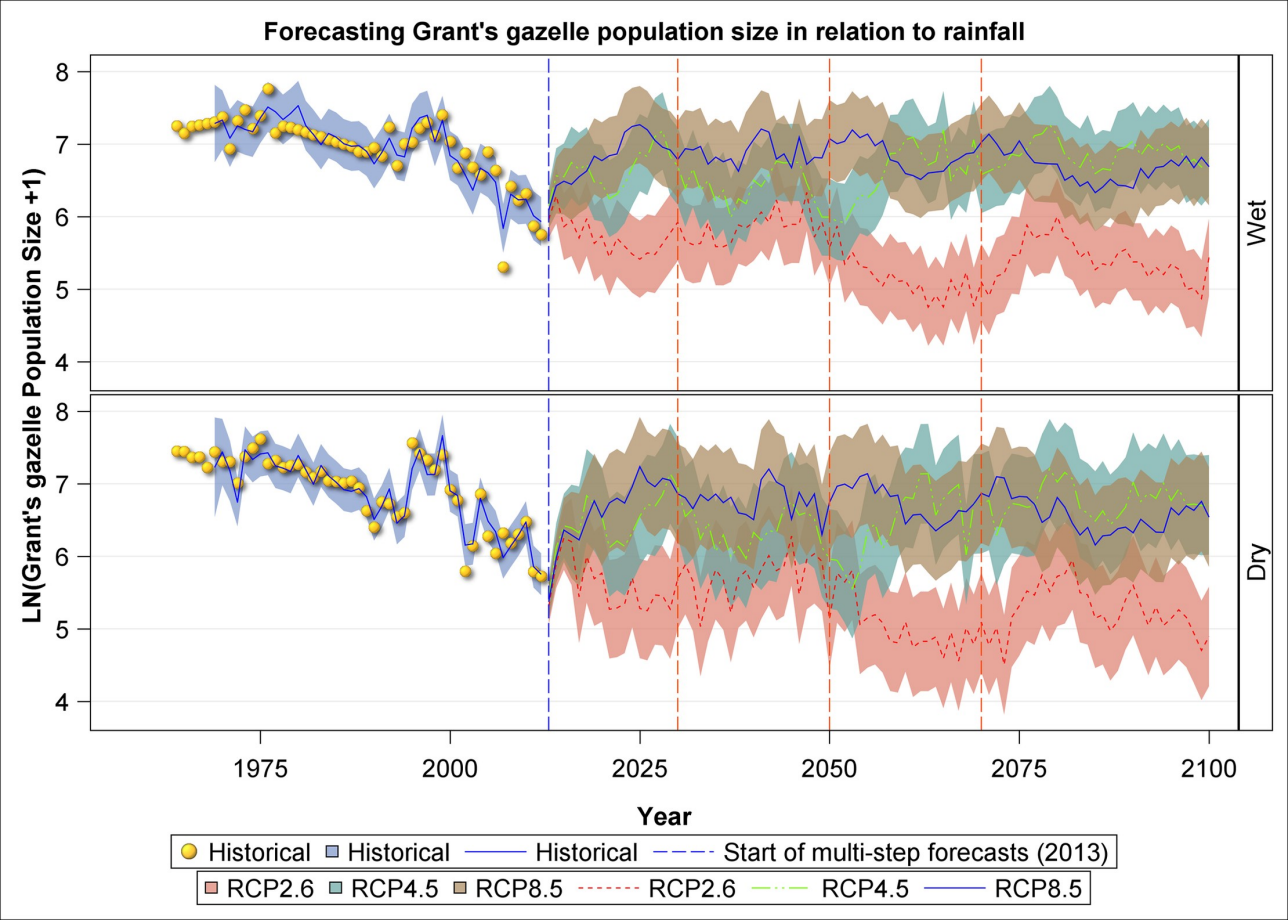
Historic and projected population size of Grant’s gazelle in the Ngorongoro Crater during the wet and dry seasons based on the three climate change scenarios: RCP 2.6, RCP 4.5 and RCP 8.5.

As with Thomson’s gazelles, the projected population trajectories for Grant’s gazelle show marked and sustained oscillations (Fig 11). Despite these persistent oscillations, Grant’s gazelle numbers will likely remain lower than the historically attained peak numbers around 1974–1976. Moreover, the declining trend in Grant’s gazelle numbers is projected to be replaced by an increasing trend after some time under the RCP 4.5 and 8.5 scenarios for both seasons. Even, so Grant’s gazelle numbers, are less likely to increase up to the greatest historically recorded numbers around 1974–1976 (Fig 11). Consistent with their small body size and selective grazing, Grant’s gazelles will also likely flourish the best under RCP 8.5 with the least rainfall, intermediately under RCP 4.5, and the worst under RCP 2.6 (Fig 11).

## Discussion

### Rainfall

Drought is a recurrent feature of the Ngorongoro Conservation Area. The annual rainfall shows evident persistent and deterministic quasi-periodic oscillation with a cycle period of about 5 years. Oscillations in the wet and dry season rainfall were stochastic and transient. The quasi-cyclic oscillations in annual, wet and dry season rainfall were statistically significant. The oscillations are associated with recurrent severe droughts that cause food scarcity and hence nutritional stress for the large herbivores. The wet season rainfall increased systematically in Ngorongoro between 1964 and 2014 but the annual or dry season rainfall did not increase. The oscillations in rainfall imply that the large herbivores are exposed to above average food supply for about 2.5 years and to below average food supply for the subsequent 2.5 years. The rainfall patterns also imply that portions of the crater may be waterlogged or flooded during the high rainfall years. High rainfall supports above-average production of plant biomass. But the forage produced during high rainfall years is likely to be of low quality due to the dilution of plant nutrients. Predation risk for herbivores is also likely to rise due to poor visibility associated with tall grass growth during periods of high rainfall [88].

### Historic herbivore population dynamics

Temporal variation in herbivore numbers in the crater followed four general patterns. First, buffalo, elephant and ostrich numbers increased significantly in the crater from 1974–2012. The transition of the crater grasslands to a majority of the area being mid to tall-grass would have favored Cape buffalo reproduction and survivorship. The increase in ostrich and elephant numbers in both seasons became more marked after the severe 1993 drought. Second, the overall average number of zebra in the crater appeared stable whereas numbers of the other eight species declined substantially between 1974 and 2012 relative to their peak numbers during 1974–1976. Third, numbers of both gazelles, eland, kongoni, waterbuck (wet season only) and Black rhino declined significantly in the crater in both seasons following the removal of the Maasai and their cattle from the crater in 1974. The decline in Black rhino is mainly attributed to poaching in the 1970's and 1980's which reduced the population to 10 individuals [31]. Fourth, wildebeest numbers decreased in the crater between 1974 and 2012 but this decrease was not statistically significant. In addition, some herbivore species were consistently more abundant inside the crater during the wet than the dry season. This pattern was most evident for the large herbivore species requiring bulk forage, comprising buffalo, eland, elephant and Black rhino. The latter may spend less time in the swamps and the forest during the wet season and may be easier to count.

### Herbivore biomass

Despite the significant changes in the population sizes of individual species in the crater, the total herbivore biomass remained relatively stable from 1963 to 1974 and from 1974–2012, implying that the crater has a stable multi-herbivore community. There is a tendency towards a higher biomass during the wet season, but it is not significant. Total wild herbivore biomass has not been significantly affected by the removal of the pastoralists and their livestock. The change in the grassland structure from mainly short grasses to mid to tall grasses after the removal of the Maasai and their livestock may have enhanced the forage availability for Cape buffalo, a large-bodied ruminant. The biomass of buffalo had the most dramatic increase post 1974 to become a major constituent of the total large herbivore biomass after the elimination of cattle from the crater in 1974. A similar increase in buffalo numbers at the expense of small and medium herbivores has also been documented for Nairobi and Lake Nakuru National Parks in Kenya [73,74].

### Relationship between herbivore population size and rainfall

Rainfall significantly influenced herbivore abundance in Ngorongoro Crater and this influence varied with species and season and partly reflect functional distinctions between the species based on their life-history traits (body size, gut morphology) or life-history strategies (feeding and foraging styles). Herbivores responded to rainfall variation in three different ways in both seasons. In the wet season, numbers of herbivore species either decreased (wildebeest, eland, kongoni, waterbuck and Black rhino), increased (zebra, buffalo, ostrich and elephant) or increased up to intermediate levels of rainfall and then decreased with further increase in rainfall (both gazelles and warthog). Similarly, in the dry season the numbers of the herbivore species either decreased (both gazelles and waterbuck), increased (wildebeest, zebra, buffalo, ostrich and warthog) or increased up to intermediate levels of rainfall and then decreased with further increase in rainfall (eland, kongoni, elephant and Black rhino).

### Forecasted herbivore population dynamics under projected climate change scenarios

The projected population trends suggest strong interspecific contrasts regarding the scenario under which each species will likely perform best but broad similarities exist between seasons for each scenario. Except for buffalo whose numbers appear to approach asymptotes, population trajectories for wildebeest, zebra and both gazelles exhibit pronounced and sustained oscillatory dynamics, reflecting similar oscillations in the projected rainfall, consistent with expectation. The projected population trajectories for buffalo and zebra suggest that both species will be most abundant in the crater under the RCP 2.6 scenario, intermediate under RCP 4.5 and least abundant under RCP 8.5 in both seasons. This supports the prediction that large-sized herbivores dependent on bulk, low-quality forage should prosper under the wet and cooler conditions expected under RCP 2.6. This is expected since buffalo is a large-sized bulk grazer and zebra is a large-sized non-ruminant able to process large quantities of low-quality forage expected to be most abundant under the wetter and cooler conditions anticipated under RCP 2.6 relative to RCP 4.5 and 8.5. Moreover, for both buffalo and zebra, the projected trajectories are generally similar between the RCP 4.5 and 8.5 scenarios for both seasons.

By contrast, the wildebeest that requires short, green grass is anticipated to be more abundant under the RCP 4.5 and 8.5 scenarios than under the RCP 2.6 scenario with wetter conditions in the wet season. In the more arid dry season conditions, wildebeest should however thrive better under the moister RCP 2.6 scenario than under RCPs 4.5 and 8.5.

Trajectories for both gazelles suggest that both species will be most abundant under RCP 8.5 with the lowest average rainfall, intermediate under RCP 4.5 with intermediate rainfall and least abundant under RCP 2.6 with the highest rainfall. This agrees with the prediction that small-sized herbivores that require high-quality forage should perform better under the comparatively arid and warmer conditions anticipated under RCP 4.5 and 8.5 than under RCP 2.6. This is consistent with the preference of both species for high-quality, short grasses and forbs. For both gazelles numbers will likely increase from about 2050–2060 to 2100 under RCP 4.5. Also, for both gazelles, the projections suggest persistent and similar population oscillations between both seasons under each of the three scenarios. The oscillations suggest extended periods of population decline followed by increase for both gazelles in both seasons. We reiterate that these projections are based solely on rainfall influences on large herbivore population dynamics, yet the dynamics of large herbivores are often influenced by a multitude of other factors.

### Predation

The major predators for large herbivores in Ngorongoro Crater are lions (*Panthera leo*) and spotted hyenas (*Crocuta crocuta*). These species, their population dynamics and feeding ecology have been studied in the crater since the 1960’s [25–29, 89–91].

Research on spotted hyenas in the crater indicates that their population size is positively correlated with their prey population size, i.e., wildebeest, zebra, Thomson’s and Grant’s gazelles [91]. Accordingly, Höner et al [91] attribute the decline in the hyena population size from the 1960’s to 1996 to the concurrent decline in their prey populations. From 1996 to 2002, the major predictor for the spotted hyena population increase was the increase in their prey population. Subsequently there was a reduction and then recovery of the population during an outbreak of *Streptococcus equi ruminatorum* in 2001 to 2003 [92]. In the short-term, the bacterial infection had a top-down impact on sex and age classes that had relatively poor nutrition. In the longer-term after the disease perturbation, the reduced population growth was due to lower juvenile survival. By 2008 the population had recovered and was approximately 450 [92] and in 2012 the population was estimated at 508 of which 364 were adults (pers comm Höner 2018).

Long term research on lions in the Ngorongoro Crater [28,29,90] indicates that the lion population may not be food limited but that it may be limited by weather extremes (high rainfall/drought) correlated with disease outbreaks and pest infestations (Canine distemper virus and biting *Stomoxys* flies). The resulting mortality is exacerbated by pride takeovers and infanticide. A severe infestation of *Stomoxys* flies in 1962 reduced the lion population of approximately 60 to 75 individuals to nine females and one male that were subsequently joined by seven immigrating males in 1975 [28]. This severe population reduction may have been a ‘bottleneck’ and the current population may be based on 15 founders [28]. The population rose to a high of 124 lions in 1983, but by 1991 there were 75 to 100 lions, and numbers dropped to 29 in 1998 [28,29]. The lion population may be density dependent since it has had positive reproductive performance when the population has been less than 60 individuals and has had negative reproductive performance when the population was more than 60 individuals. From 1994 to 2004, the population had not had reduced reproductive performance. Kissui and Packer [29] attribute the declines in the lion population to disease outbreaks that correlated with extreme weather events that occurred in 1962, 1994, 1997, and 2001. During 2000/2001 there was a decrease in the lion population due to death (*Stomoxys* flies) and emigration [91].

## Conclusions

Ngorongoro Crater has an annual rainfall cycle period of about 5 years. Oscillations in annual, wet and dry season rainfall were statistically significant. The oscillations are associated with recurrent severe droughts that cause food scarcity and hence nutritional stress for the large herbivores. Rainfall oscillations imply that large herbivores are exposed to above average food supply for about 2.5 years and to below average food supply for the subsequent 2.5 years. High rainfall supports above-average production of plant biomass, which may be of low quality due to the dilution of plant nutrients by fibres [3, 49]. Rainfall variation therefore can impact herbivore population size by modulating the fibre content of available forage. Increases in vegetation biomass and fibre will tend to favour large ruminant and non-ruminant herbivores. Large ruminant herbivores can subsist on a diet rich in fibre but small ruminant herbivores need to feed selectively on rapidly digestible, low-fibre foods [49].

In 1974 there was a perturbation in that resident Maasai and their livestock were removed from the Crater. Vegetation maps from before and after the removal of pastoralists and their livestock indicate that major changes in vegetation structure occurred. The 1967/68 map versus the 1995 vegetation map shows that there was a significant change in the vegetation structure of the crater floor, such that there was a decrease in the availability of short grasses and an increase in medium and tall grassland. However, there is no information on the structure and composition of the grassland vegetation from 1967 to 1995. The available vegetation maps can only provide snapshots of the potential long-term change in vegetation structure.

Temporal variation in herbivore numbers in the crater followed four general patterns. First, buffalo, elephant and ostrich numbers increased significantly in the crater from 1974–2012. Second, the overall average number of zebra in the crater appeared stable whereas numbers of the other eight species declined substantially between 1974 and 2012 relative to their peak numbers during 1974–1976. Third, numbers of both gazelles, eland, kongoni, waterbuck (wet season only) and Black rhino declined significantly in the crater in both seasons following the removal of the Maasai and their cattle from the crater in 1974. The decline in Black rhino is mainly attributed to poaching in the 1970's and 1980's. Fourth, wildebeest numbers decreased in the crater between 1974 and 2012 but this decrease was not statistically significant. In addition, some herbivore species were consistently more abundant inside the crater during the wet than the dry season. This pattern was most evident for the large herbivore species requiring bulk forage, comprising buffalo, eland, elephant and Black rhino. The latter may spend less time in the swamps and the forest during the wet season and may be easier to count. Even with a change in grassland structure, total herbivore biomass remained relatively stable from 1963 to 2012, implying that the crater has a stable multi-herbivore community.

Rainfall significantly influenced herbivore abundance in Ngorongoro Crater and this influence varied with species and season. Herbivores responded to rainfall variation in three different ways in both seasons. In the wet season, numbers of herbivore species either decreased (wildebeest, eland, kongoni, waterbuck and black rhino), increased (zebra, buffalo, ostrich and elephant) or increased up to intermediate levels of rainfall and then decreased with further increase in rainfall (both gazelles and warthog). Similarly, in the dry season the numbers of the herbivore species either decreased (both gazelles and waterbuck), increased (wildebeest, zebra, buffalo, ostrich and warthog) or increased up to intermediate levels of rainfall and then decreased with further increase in rainfall (eland, kongoni, elephant and Black rhino).

The relationships established between the time series of historic animal counts in the wet and dry seasons and lagged wet and dry season rainfall series were used to forecast the likely future trajectories of the wet and dry season population size for each species under three alternative climate change scenarios. They suggest strong interspecific contrasts regarding the scenario under which each species will likely perform best but broad similarities exist between seasons for each scenario and species.

Disease is an important perturbation in the population dynamics of lions and spotted hyenas and potentially of Black rhino, buffalo and other herbivores. Tick borne diseases, in particular, can potentially be managed with systematic burning of some grassland areas. It would be useful to examine in detail the interactions between rainfall, herbivore natality, mortality and population size, disease outbreaks, spotted hyena and lion predation on preferred prey species and predator population trends.

## Supporting information

S1 Data Total monthly rainfall (in mm) and the total annual rainfall recorded at the Ngorongoro Conservation Area Authority Headquarters located on the southern crater rim from 1963 to 2014. (XLSX)

S2 Data The count totals for each of the 12 most common large herbivore species counted during the wet and the dry seasons in the Ngorongoro Crater from 1964 to 2012. The agency or organization (source) that did the survey and the method (Method) used to count animals are also provided. NCA = Ngorongoro Conservation Authority; TWA = Turner and Watson; WAT = Watson; TBE = Turner and Bell; TLA = Turner and Lamprey; DML = Des Meules and Lemeiux; MWE = Mweka College of African Wildlife Management; ECO = Ecosystems Ltd; SEM = Serengeti Ecological Monitoring Programme and NEM = Ngorongoro Ecological Monitoring Programme.
(XLSX)

S3 Data The count totals for each of the 12 most common large herbivore species counted during the wet and the dry seasons in the Ngorongoro Crater from 1964 to 2012. The missing values were imputed using a state space model, separately for each species and season combination.
(XLSX)

S4 Data The logarithm of the observed (historical) and predicted population size for each of the five most common herbivore species for the wet and dry season and the 95% pointwise lower and upper prediction confidence limits for 1964 to 2012 for each of the three climate change scenarios: RCP 2.6, RCP 4.5 and RCP 8.5. The logarithm of the forecasted population size is also provided for each of the five most abundant herbivore species for 2013 to 2100 under each of the three climate change scenarios: RCP 2.6, RCP 4.5 and RCP 8.5.
(XLSX)

S1 Table Parameter estimates for the bivariate VARMAX (2,2,5) model for the five most abundant herbivore species in the dry and wet seasons in the Ngorongoro Crater, Tanzania, during 1963–2012. Model selection was based on information theory so no effort has been made to remove insignificant coefficients. By restricting a few of the highly insignificant coefficients to be zero, many of the apparently insignificant coefficients become significant.
(XLSX)

S2 Table Roots of AR characteristic polynomials for the bivariate model for the five most abundant herbivore species in the dry and wet seasons in the Ngorongoro Crater, Tanzania, during 1963–2012. The modulus of the roots of its AR polynomial should be less than 1 for a time series to be stationary.
(XLSX)

S3 Table Roots of the MA characteristic polynomials for the bivariate model for the five most abundant herbivore species in the dry and wet seasons in the Ngorongoro Crater, Tanzania, during 1963–2012. (XLSX)

S4 Table Portmanteau Test for Cross Correlations of Residuals from the bivariate VARMAX(2,2,5) model for the five most abundant herbivore species in the dry and wet seasons in the Ngorongoro Crater, Tanzania, during 1963–2012. The results show tests for white noise residuals based on the cross correlations of the residuals. Insignificant test results show that we cannot reject the null hypothesis that the residuals are uncorrelated.
(XLSX)

S5 Table Univariate model ANOVA diagnostics for the five most abundant herbivore species in the dry and wet seasons in the Ngorongoro Crater, Tanzania, during 1963–2012. The results show that each model is significant.
(XLSX)

S6 Table Univariate Model White Noise Diagnostics for the five most abundant herbivore species in the dry and wet seasons in the Ngorongoro Crater, Tanzania, during 1963–2012. The results show tests of whether the residuals are correlated and heteroscedastic. The Durbin-Watson test statistics test the null hypothesis that the residuals are uncorrelated. The Jarque-Bera normality test tests the null hypothesis that the residuals are normally distributed. The F statistics and their *p*-values for ARCH(1) disturbances test the null hypothesis that the residuals have equal covariances.
(XLSX)

S7 Table Univariate AR Model Diagnostics for the five most abundant herbivore species in the dry and wet seasons in the Ngorongoro Crater, Tanzania, during 1963–2012. The *F* statistics and their *p*-values for AR(1), AR(1,2), AR(1,2,3) and AR(1,2,3,4) models of residuals test the null hypothesis that the residuals are uncorrelated.
(XLSX)

S8 Table Classification of years and seasons into extreme drought, severe drought, moderate drought, normal, wet, very wet and extremely wet years or seasons using percentiles of the frequency distributions of the total annual, wet season or dry season rainfall recorded at the Ngorongoro Conservation Area Headquarters from 1963 to 2014. The percentile of rainfall components used to delineate the classes are also provided.
(XLSX)

S9 Table The estimated frequency, period, periodogram, spectral density, co-spectra, quadrature, squared coherence, amplitude and phases of the oscillations in the annual, wet and dry season rainfall components for the Ngorongoro Crater during 1963–2014. (XLSX)

S10 Table The estimated variances of the disturbance terms, the variances of the irregular components, damping factors and periods of the cycles in the annual, wet and dry season rainfall components recorded for the Ngorongoro Crater during 1963–2014. (XLSX)

S11 Table Significance analysis of the rainfall model components (based on the final state). (XLSX)

S12 Table The expected population size of each of the 12 most common large wild herbivore species in 1964, 1974 and 2012 and the difference between the estimates for 1964 and 1974 and 1974 and 2012 and tests of significance of the differences based on constructed penalized cubic B-splines. (XLSX)

S13 Table Selection of the rainfall component, moving average and functional form of the relationship between population size and the moving average component for each of the 12 most common large herbivore species based on the corrected Akaike Information Criterion (AICc). Only models with delta AICc no more than 4 are shown. Model selection was carried out separately for the wet and dry season counts for each species.
(XLSX)

S14 Table Parameters estimates, their standard errors and *t* tests of whether the parameters are significantly different from zero for the AICc-selected best models relating population size and moving average rainfall, for the wet and season counts, for the 12 most common large herbivore species in the Ngorongoro Crater. (XLSX)

S15 Table Changes in vegetation structure from 1966/67 to 1995. (DOCX)

S1 Text SAS code used to analyze the rainfall data for the Ngorongoro Conservation Area Headquarters. (DOCX)

S2 Text SAS code used to model trends in the animal counts, relate the counts to rainfall and project population dynamics to 2013–2100. (DOCX)

S1 Fig Temporal variation in the original (blue vertical needles) and smoothed (red solid curve) total monthly rainfall in the Ngorongoro Crater from 1963 to 2014. (PNG)

S2 Fig Percentiles of the total annual, dry and wet season rainfall components. The percentiles are used to classify years or seasons as extreme, severe or moderate drought years or seasons, normal, wet, very wet or extremely wet years or seasons as described in the text.
(PNG)

S3 Fig Spectral density versus period of cycles (in years) for a) annual rainfall, b) wet season rainfall, and c) dry season rainfall based on rainfall recorded at the Ngorongoro Conservation Authority Headquarters from 1963 to 2014. A large value of spectral density means that the corresponding cycle period has greater support in the data.
(PDF)

S4 Fig Smoothed cycles and trends based on the structural time series analysis versus the year of observation for the standardized annual (annualstd), wet season (wetstd) and dry season (drystd) rainfall components for the Ngorongoro Crater for 1963–2014. (PDF)

S5 Fig Projected total annual rainfall, average maximum and minimum temperatures for Ngorongoro Crater in Tanzania under three climate change scenarios (RCP 2.6, RCP 4.5 and RCP 8.5) for the period 2006–2100. (PNG)

S1 File (JPG)
