# Supplementary material for: Long-term historical and projected herbivore population dynamics in Ngorongoro crater, Tanzania
Source: PLoS One. 2020 Mar 10;15(3):e0212530. doi: 10.1371/journal.pone.0212530 (PMC7064247; doi:10.1371/journal.pone.0212530)
Supplement: S15 Table — (DOCX) [file pone.0212530.s019.docx]

**S15 Table**. **Changes in vegetation structure from 1966/67 to 1995.**

| Major Herbaceous Species (ha) | | |
| --- | --- | --- |
| Herlocker | | Chuwa |
| Short | 20868 | 10626 |
| Mid | 2110 | 9540 |
| Mid-tall | 5794 | 4506 |
| Tall | 1377 | 4227 |
| Secondary Herbaceous Species (ha) | | |
| Short | 18584 | 4794 |
| Mid | 2812 | 3905 |
| Mid-tall | 2004 | 9096 |
| Tall | 1317 | 2258 |
