# Supplementary material for: Long-term historical and projected herbivore population dynamics in Ngorongoro crater, Tanzania
Source: PLoS One. 2020 Mar 10;15(3):e0212530. doi: 10.1371/journal.pone.0212530 (PMC7064247; doi:10.1371/journal.pone.0212530)
Supplement: S1 Text — (DOCX) [file pone.0212530.s020.docx]

**PROC** **IMPORT** OUT= WORK.rainfall

DATAFILE= "F:\joseph_2009\Ngorongoro\data\NCA hqts rainfall 1963_2014_4.xlsx"

DBMS=EXCEL REPLACE;

RANGE="Sheet1$";

GETNAMES=YES;

MIXED=NO;

SCANTEXT=YES;

USEDATE=YES;

SCANTIME=YES;

**RUN**;

**data** rain_month1; set rainfall;

array rainxx[**12**] Jan--Dec;

do month=**1** to **12**;

rain=rainxx[month] ;

output;

end;

keep Year month rain;

**run**;

**data** rain_month2; set rain_month1;

time=_n_;

month2=month;

**run**;

/*proc univariate data=rain_month2;

var rain rainsq rainlog;

histogram rain rainsq rainlog/normal;

run;*/

**Data** rain2; set rainfall;

dry=JUN+JUL+AUG+SEP+OCT;

wet1=NOV+ DEC;

Wet11=lag1(wet1);

wet2= JAN+FEB+MAR+APR+MAY;

wet=wet11+wet2;

annual=wet+dry;

mavdry1=dry;

mavdry2=(mavdry1+lag1(mavdry1))/**2**;

mavdry3=(**2***mavdry2+lag2(mavdry1))/**3**;

mavdry4=(**3***mavdry3+lag3(mavdry1))/**4**;

mavdry5=(**4***mavdry4+lag4(mavdry1))/**5**;

mavdry6=(**5***mavdry5+lag5(mavdry1))/**6**;

mavwet1=wet;

mavwet2=(mavwet1+lag1(mavwet1))/**2**;

mavwet3=(**2***mavwet2+lag2(mavwet1))/**3**;

mavwet4=(**3***mavwet3+lag3(mavwet1))/**4**;

mavwet5=(**4***mavwet4+lag4(mavwet1))/**5**;

mavwet6=(**5***mavwet5+lag5(mavwet1))/**6**;

mavannual1=annual;

mavannual2=(mavannual1+lag1(mavannual1))/**2**;

mavannual3=(**2***mavannual2+lag2(mavannual1))/**3**;

mavannual4=(**3***mavannual3+lag3(mavannual1))/**4**;

mavannual5=(**4***mavannual4+lag4(mavannual1))/**5**;

mavannual6=(**5***mavannual5+lag5(mavannual1))/**6**;

drop Jan--Dec wet11 wet1 wet2 total comments;

**run**;

**proc** **means** data=rain2 mean std min max;

var dry wet annual;

output out=means (drop=_type_ _freq_) ;

**run**;

**proc** **stdize** data=rain2 method=std pstat out=rain2std(keep=year dry wet annual mavannual2 mavdry2 mavwet2 mavannual3 mavdry3 mavwet3 mavannual4 mavdry4 mavwet4

mavannual5 mavdry5 mavwet5 mavannual6 mavdry6 mavwet6

rename=(dry=drystd wet=wetstd

annual=annualstd mavannual2=mavannualstd2 mavdry2=mavdrystd2 mavwet2=mavwetstd2 mavannual3=mavannualstd3 mavdry3=mavdrystd3

mavwet3=mavwetstd3 mavannual4=mavannualstd4 mavdry4=mavdrystd4 mavwet4=mavwetstd4 mavannual5=mavannualstd5 mavwet5=mavwetstd5 mavdry5=mavdrystd5

mavannual6=mavannualstd6 mavdry6=mavdrystd6 mavwet6=Mavwetstd6 ));

var annual wet dry mavannual2 mavdry2 mavwet2 mavannual3 mavdry3 mavwet3 mavannual4 mavdry4 mavwet4 mavannual5 mavdry5 mavwet5 mavannual6 mavdry6 mavwet6 ;

**run**;

**proc** **univariate** data=rain2std;

var annualstd wetstd drystd;

output out=percentiles pctlpts=**10** **25** **40** **75** **90** **95** **100** pctlpre= a w d;

**run**;

**data** percentiles2; set percentiles;

index=**1**;

**run**;

**data** rain2std; set rain2std;

index=**1**;

**run**;

**proc** **sort** data=percentiles2;

by index;

**run**;

**Proc** **sort** data=rain2std;

by index;

**run**;

**Data** rain2xx;

merge rain2std percentiles2;

by index;

format year **4.0**;

**run**;

**proc** **means** data=rain2xx nway noprint;

class year;

var annualstd wetstd drystd mavannualstd2 mavwetstd2 mavdrystd2 mavannualstd3 mavwetstd3 mavdrystd3 mavannualstd4 mavwetstd4 mavdrystd4 mavannualstd5 mavwetstd5 mavdrystd5 mavannualstd6 mavwetstd6 mavdrystd6

d10 d25 d40 d75 d90 d95 d100

a10 a25 a40 a75 a90 a95 a100

w10 w25 w40 w75 w90 w95 w100

;

output out=rain3xx(drop=_type_ _freq_) mean=;

**run**;

**data** droughts; set rain2xx;

if annualstd<=a10 then do; a='extreme '; percent=**10**; end;

else if a10<annualstd<=a25 then do; a='severe' ; percent=**25**; end;

else if a25<annualstd<=a40 then do; a='moderate'; percent=**40**; end;

else if a40<annualstd<=a75 then do; a='normal'; percent=**75**; end;

else if a75<annualstd<=a90 then do; a='wet'; percent=**90**; end;

else if a90<annualstd<=a95 then do; a='vwet'; percent=**95**; end;

else if a95<annualstd<=a100 then do; a='extwet'; percent=**100**; end;

if wetstd<=w10 then do; w='extreme '; percent=**10**; end;

else if w10<wetstd<=w25 then do; w='severe'; percent=**25**; end;

else if w25<wetstd<=w40 then do; w='moderate'; percent=**40**; end;

else if w40<wetstd<=w75 then do; w='normal'; percent=**75**; end;

else if w75<wetstd<=w90 then do; w='wet'; percent=**90**; end;

else if w90<wetstd<=w95 then do; w='vwet'; percent=**95**; end;

else if w95<wetstd<=w100 then do; w='extwet'; percent=**100**; end;

if drystd<=d10 then do; d='extreme'; percent=**10**; end;

else if d10<drystd<=d25 then do; d='severe'; percent=**25**; end;

else if d25<drystd<=d40 then do; d='moderate'; percent=**40**; end;

else if d40<drystd<=d75 then do; d='normal'; percent=**75**; end;

else if d75<drystd<=d90 then do; d='wet'; percent=**90**; end;

else if d90<drystd<=d95 then do; d='vwet'; percent=**95**; end;

else if d95<drystd<=d100 then do; d='extwet'; percent=**100**; end;

**run**;

**data** droughts_floods;

set droughts(rename=(annualstd=rain a=class) in=a1)

droughts(rename=(wetstd=rain w=class) in=w1)

droughts(rename=(drystd=rain d=class) in=d1);

if a1 then component='annual';

if w1 then component='wet';

if d1 then component='dry';

if year< **1977** then period ='earlier';

else period='recent';

keep year rain class component period percent;

**run**;

**Proc** **sort** data=droughts_floods;

by component year;

**run**;

**proc** **means** data= droughts_floods nway noprint;

class period component class;

var rain;

output out=classes(drop=_type_ _freq_) n=;

**run**;

/*----Wet season and annual periodicity=2.94,dry seaosn=2.65.*/;

**data** rain4xx; set rain3xx;

y2=**0**;

**run**;

%let outdir=F:\joseph_2009\Ngorongoro\Graphs;

**proc** **template**;

define style myfont6;

parent=styles.printer;

style

GraphFonts /

'GraphDataFont' = ("Helvetica",**16**pt)

'GraphUnicodeFont' = ("Helvetica",**14**pt)

'GraphValueFont' = ("Helvetica",**14**pt)

'GraphLabelFont' = ("Helvetica",**14**pt,bold)

'GraphFootnoteFont' = ("Helvetica",**6**pt,bold)

'GraphTitleFont' = ("Helvetica",**14**pt,bold)

'GraphAnnoFont' = ("Helvetica",**6**pt);

*style GraphBackground / backgroundcolor=white;

*style body /background=white;

*class graphbackground / color=white;

*class graphwalls / color=white;

*class graphgridlines / contrastcolor=black;

end;

**run**;

options orientation=portrait nodate nonumber;

goptions reset=all ;

/* This step uses multiple PROC SGPLOT procedures */

/* to write multiple PNG files to disk. Note that */

/* this step uses the IMAGENAME= option on the ODS */

/* GRAPHICS ON statement to name each PNG file */

/* that is written to disk. */

*ods rtf file="&outdir.\LNNPfont.rtf" style=myfont;

ods results off;

ods listing close;

ods html path="&outdir" (url=none) file='sastest.html'

image_dpi=**300** style=myfont6;

ods graphics on / imagename='Rainfall extremes'

width=**24** cm height=**20** cm;

**proc** **sgpanel** data=droughts_floods noautolegend;

panelby Component/ novarname layout=panel columns=**1** rows=**3** spacing=**2** uniscale=all;

needle x=year y=percent/ lineattrs=(color=firebrick pattern=**1** thickness=**14**) transparency=**0.5**;

*bubble x=Period y=s_02 size=size / dataskin=gloss fill fillattrs=(color=firebrick) transparency=0.0 ;

title "Rainfall extremes in Ngorongoro Crater, Tanzania" ;

rowaxis label='Percentiles' values=(**0** to **100** by **10**) grid;

colaxis label='Year';

*where period<=10;

**run**;

**proc** **spectra** data=rain2 out=rain_spectra coef s ph p a k whitetest adjmean center;

var annual wet Dry ;

weights parzen ;

**run**;

**data** rain_spectra2; set Rain_spectra;

size=**0.1**;

**run**;

options nodate nonumber nobyline;

**proc** **sort** data=rain_spectra2;

by Period;

**run**;

ods graphics on / imagename='Figure_S1a'

width=**24** cm height=**12** cm;

**proc** **sgplot** data=rain_spectra2 noautolegend;

series x=period y=s_01/ /*datalabel=P_A*/;

bubble x=Period y=s_01 size=size / dataskin=gloss fill fillattrs=(color=firebrick) transparency=**0.0** ;

title "Spectrum of Annual rainfall" ;

yaxis label='Spectral density';

xaxis label='Period in years';

refline **4.67**/ axis=x label='4.67' labelpos=min labelloc=inside lineattrs=(color=blue pattern=**4** thickness=**2**);

**run**;

ods graphics on / imagename='Figure_S1b'

width=**24** cm height=**12** cm;

ods listing image_dpi=**300** style=myfont6;

**proc** **sgplot** data=rain_spectra2 noautolegend;

series x=period y=s_02/ /*datalabel=P_W*/;

bubble x=Period y=s_01 size=size / dataskin=gloss fill fillattrs=(color=firebrick) transparency=**0.0** ;

title "Spectrum of wet season rainfall" ;

yaxis label='Spectral density';

xaxis label='Period in years';

refline **4.67**/ axis=x label='4.67' labelpos=min labelloc=inside lineattrs=(color=blue pattern=**4** thickness=**2**);

**run**;

ods graphics on / imagename='Figure_S1c'

width=**24** cm height=**12** cm;

**proc** **sgplot** data=rain_spectra2 noautolegend;

series x=period y=s_03 / /*datalabel=P_D*/;

bubble x=Period y=s_03 size=size / dataskin=gloss fill fillattrs=(color=firebrick) transparency=**0.0** ;

title "Spectrum of Dry season rainfall" ;

yaxis label='Spectral density';

xaxis label='Period in years';

refline **2.47**/ axis=x label='2.47' labelpos=min labelloc=inside lineattrs=(color=blue pattern=**4** thickness=**2**);

**run**;

ods graphics on / imagename='Figure_1b'

width=**24** cm height=**12** cm;

**proc** **sgplot** data=rain4xx noautolegend;

needle x=year y=Annualstd / lineattrs=(color=blue pattern=**1** thickness=**3**) ;;

pbspline x=year y=mavannualstd5 /Lineattrs=(color=red pattern=**1** thickness=**5**) nomarkers nolegfit maxpoints=**200** Nknots=**120** degree=**2**;

refline -**1.186228857** / axis=y lineattrs=(color=black pattern=**4** thickness=**1.5**) label='10';

refline -**0.671398456** / axis=y lineattrs=(color=black pattern=**4** thickness=**1.5**) label='25';

refline -**0.278279327** / axis=y lineattrs=(color=black pattern=**4** thickness=**1.5**) label='40';

refline **0.5930740236** / axis=y lineattrs=(color=black pattern=**4** thickness=**1.5**) label='75';

refline **1.0565704184** / axis=y lineattrs=(color=black pattern=**4** thickness=**1.5**) label='90';

refline **2.2933887723** / axis=y lineattrs=(color=black pattern=**4** thickness=**1.5**) label='95';

refline **2.9319175677** / axis=y lineattrs=(color=black pattern=**4** thickness=**1.5**) label='100';

title "Annual rainfall (mean = 932.9 mm, SD = 302.9 mm)" ;

yaxis label='Standardized rainfall' offsetmin=**0.1** ;

xaxis values=(**1963** to **2017** by **6**);

y2axis display= (novalues) label='Percentiles';

Series x=year y=y2 /y2axis transparency=**1** ;

inset 'b' /noborder noborder position=Bottomleft textattrs=(size=**30**);

**run**;

**quit**;

ods graphics on / imagename='Figure_1c'

width=**24** cm height=**12** cm;

**proc** **sgplot** data=rain4xx noautolegend;

needle x=year y=wetstd / lineattrs=(color=blue pattern=**1** thickness=**3**) ;;

pbspline x=year y=mavwetstd5 /Lineattrs=(color=red pattern=**1** thickness=**5**) nomarkers nolegfit maxpoints=**200** Nknots=**120** degree=**2**;

refline -**1.100056721** / axis=y lineattrs=(color=black pattern=**4** thickness=**1.5**) label='10';

refline -**0.71172274** / axis=y lineattrs=(color=black pattern=**4** thickness=**1.5**) label='25';

refline -**0.397277532**/ axis=y lineattrs=(color=black pattern=**4** thickness=**1.5**) label='40';

refline **0.6700976163** / axis=y lineattrs=(color=black pattern=**4** thickness=**1.5**) label='75';

refline **1.098552198** / axis=y lineattrs=(color=black pattern=**4** thickness=**1.5**) label='90';

refline **2.4186871303** / axis=y lineattrs=(color=black pattern=**4** thickness=**1.5**) label='95';

refline **2.7914743785**/ axis=y lineattrs=(color=black pattern=**4** thickness=**1.5**) label='100';

title "Wet season rainfall (mean = 848.0 mm, SD = 299.1 mm)";

yaxis label='Standardized rainfall' offsetmin=**0.1**;

xaxis values=(**1963** to **2017** by **6**);

y2axis display= (novalues) label='Percentiles';

Series x=year y=y2 /y2axis transparency=**1** ;

inset 'c' /noborder position=Bottomleft textattrs=(size=**30**);

**run**;

**quit**;

ods graphics on / imagename='Figure_1d'

width=**24** cm height=**12** cm;

**proc** **sgplot** data=rain4xx noautolegend;

where year<=**2014**;

needle x=year y=drystd / lineattrs=(color=blue pattern=**1** thickness=**3**) ;

pbspline x=year y=mavdrystd2 /Lineattrs=(color=red pattern=**1** thickness=**5**) nomarkers nolegfit maxpoints=**200** Nknots=**120** degree=**2**;

refline -**0.874788248** / axis=y lineattrs=(color=black pattern=**4** thickness=**1.5**) label='10';

refline -**0.640047151** / axis=y lineattrs=(color=black pattern=**4** thickness=**1.5**) label='';

refline -**0.469744787** / axis=y lineattrs=(color=black pattern=**4** thickness=**1.5**) label='40';

refline **0.4807265159** / axis=y lineattrs=(color=black pattern=**4** thickness=**1.5**) label='75';

refline **1.2087307665** / axis=y lineattrs=(color=black pattern=**4** thickness=**1.5**) label='90';

refline **2.1875857967** / axis=y lineattrs=(color=black pattern=**4** thickness=**1.5**) label='95';

refline **3.935102849** / axis=y lineattrs=(color=black pattern=**4** thickness=**1.5**) label='100';

title "Dry season rainfall (mean = 85.5 mm, SD = 65.2 mm)";

yaxis label='Standardized rainfall' offsetmin=**0.10** ;

y2axis display= (novalues) label='Percentiles';

Series x=year y=y2 /y2axis transparency=**1** ;

xaxis values=(**1963** to **2017** by **6**);

inset 'd' /noborder position=Bottomleft textattrs=(size=**30**);

**run**;

**quit**;

/*proc print data=percentiles;

var d10 d25 d40 d75 d90 d95 d100;

run;

*/

/*----------------------------Plotting monthly rainfall distribution-----------------*/;

**data** rain3; set rainfall;

array NG [**12**] Jan--Dec;

do Month=**1** to **12**;

rain=NG[month];

output;

end;

keep year month rain;

**run**;

**Proc** **means** data=rain3 nway noprint;

class month;

var rain;

output out=means(drop=_type_ _freq_) mean=;

**run**;

**proc** **format** lib=work;

value monthfmt

**8**="JAN"

**9**="FEB"

**10**="MAR"

**11**="APR"

**12**="MAY"

**1**="JUN"

**2**="JUL"

**3**="AUG"

**4**="SEP"

**5**="OCT"

**6**="NOV"

**7**="DEC"

;

**run**;

**data** mean_rains; set rain3;

select(Month);

when (**1**) Month3='JAN';

when (**2**) Month3='FEB';

when (**3**) Month3='MAR';

when (**4**) Month3='APR';

when (**5**) Month3='MAY';

when (**6**) Month3='JUN';

when (**7**) Month3='JUL';

when (**8**) Month3='AUG';

when (**9**) Month3='SEP';

when (**10**) Month3='OCT';

when (**11**) Month3='NOV';

when (**12**) Month3='DEC';

otherwise;

end;

**run**;

**data** mean_rains2; set mean_rains;

select(Month3);

when ('JAN') Month4=**8**;

when ('FEB') Month4=**9**;

when ('MAR') Month4=**10**;

when ('APR') Month4=**11**;

when ('MAY') Month4=**12**;

when ('JUN') Month4=**1**;

when ('JUL') Month4=**2**;

when ('AUG') Month4=**3**;

when ('SEP') Month4=**4**;

when ('OCT') Month4=**5**;

when ('NOV') Month4=**6**;

when ('DEC') Month4=**7**;

otherwise;

end;

format Month4 monthfmt4.;

mean_rain=**78.3**;

label month4=Month

rain=Mean monthly rainfall;

**run**;

**proc** **sort** data=mean_rains2;

by month4;

**run**;

%let outdir=F:\joseph_2009\Ngorongoro\Graphs;

**proc** **template**;

define style myfont7;

parent=styles.printer;

style

GraphFonts /

'GraphDataFont' = ("Helvetica",**12**pt)

'GraphUnicodeFont' = ("Helvetica",**12**pt)

'GraphValueFont' = ("Helvetica",**12**pt)

'GraphLabelFont' = ("Helvetica",**12**pt,bold)

'GraphFootnoteFont' = ("Helvetica",**6**pt,bold)

'GraphTitleFont' = ("Helvetica",**12**pt,bold)

'GraphAnnoFont' = ("Helvetica",**6**pt);

*style GraphBackground / backgroundcolor=white;

*style body /background=white;

*class graphbackground / color=white;

*class graphwalls / color=white;

*class graphgridlines / contrastcolor=black;

end;

**run**;

options orientation=portrait nodate nonumber;

goptions reset=all ;

/* This step uses multiple PROC SGPLOT procedures */

/* to write multiple PNG files to disk. Note that */

/* this step uses the IMAGENAME= option on the ODS */

/* GRAPHICS ON statement to name each PNG file */

/* that is written to disk. */

*ods rtf file="&outdir.\LNNPfont.rtf" style=myfont;

ods results off;

ods listing close;

ods html path="&outdir" (url=none) file='sastest.html'

image_dpi=**300** style=myfont7;

ods graphics on / imagename='Figure_1a'

width=**24** cm height=**12** cm;

**proc** **sgplot** data=mean_rains2 noautolegend;

vbar month4 / response=rain stat=mean limits=both limitstat=stderr fillattrs=(color=salmon);

vline month4 / response=rain stat=mean lineattrs=(color=magenta pattern=**1** thickness=**3**);

refline mean_rain / axis=y lineattrs=(color=black pattern=**4** thickness=**3**) ;

title "Mean monthly rainfall (mean = 78.3 mm, SD = 84.2 mm)";

yaxis label="Mean monthly rainfall";

xaxis offsetmin=**0.075**;

inset 'a' /noborder position=Bottomleft textattrs=(size=**30**);

**run**;

**quit**;

**data** rain3b; set rain3;

day=**1**;

month3 = mdy(put(month,**2.**),put(day,**2.**),put(year,**4.**));

format month3 monyy.;

**run**;

libname rain 'F:\joseph_2009\Ngorongoro\Results';

ods html ;

ods graphics on;

**proc** **timeseries** data=rain3b outseason=rain.season_rain outtrend=rain.trend_rain outdecomp=rain.decomp_rain outspectra=rain.spectra_rain

plot=(series ACF Corr PACF IACF residual WN DECOMP TCC TCS SIC SC TC)

seasonality=**12** ; trend;

var rain ;

Decomp orig tcc sc sic tc/mode=pseudoadd;

id month3 interval=month accumulate=avg;

spectra period s/Parzen;

**run**;

ods graphics on / imagename='Month_rain_trend'

width=**24** cm height=**18** cm;

**proc** **sgplot** data= rain.decomp noautolegend;

yaxis label='Monthly rainfall in mm';

xaxis label='Year and month';

needle x=month3 y=original /Lineattrs=(color=blue pattern=**1** thickness=**1**) baseline=**59.45** name='Mean' name='S1' legendlabel='Original';

Series x=month3 y=TC /Lineattrs=(color=red pattern=**1** thickness=**3.5**) name='S3' legendlabel='Wet';

title "Original and smoothed monthly rainfall series";

*inset 'a' /noborder position=bottomleft textattrs=(size=30);

Keylegend "S1" "S3" "S4"/location=inside across=**3** position=topleft border down=**1**;

**run**;

**quit**;

/*------Modeling cycles in annual rainfall data--*/;

ods graphics on;

ods output SmoothedCycle=annual_SmoothedCycle outliersummary=annual_outliersummary ParameterEstimates=annual_ParameterEstimates

ComponentSignificance=annual_ComponentSignificance;

**proc** **ucm** data=rain4xx;

*id year interval=year;

model annualstd;

irregular;

level ;

* slope ;

cycle plot=(filter smooth);

*cycle plot=(filter smooth);

estimate back=**0** plot=(loess panel cusum wn);

forecast back=**0** lead=**0** plot=(forecasts decomp);

**run**;

ods graphics off;

/*------Modeling cycles in wet season rainfall data--*/;

ods graphics on;

ods output SmoothedCycle=wet_SmoothedCycle outliersummary=wet_outliersummary ParameterEstimates=wet_ParameterEstimates

ComponentSignificance=wet_ComponentSignificance;

**proc** **ucm** data=rain4xx;

*id year interval=year;

model wetstd;

irregular;

level ;

slope ;

cycle plot=(filter smooth) ;

cycle plot=(filter smooth) ;

estimate back=**0** plot=(loess panel cusum wn);

forecast back=**0** lead=**0** plot=(forecasts decomp);

**run**;

ods graphics off;

/*------Modeling cycles in dry season rainfall data--*/;

ods graphics on;

ods output SmoothedCycle=dry_SmoothedCycle outliersummary=dry_outliersummary ParameterEstimates=dry_ParameterEstimates

ComponentSignificance=dry_ComponentSignificance;

**proc** **ucm** data=rain3xx;

*id year interval=year;

model drystd;

irregular;

level ;

*slope;

cycle plot=(filter smooth);

cycle plot=(filter smooth);

estimate back=**0** plot=(loess panel cusum wn);

forecast back=**0** lead=**0** plot=(forecasts decomp);

**run**;

ods graphics off;

**data** componentsignificance;

set annual_componentsignificance(in=x1) wet_componentsignificance(in=x2) dry_componentsignificance(in=x3);

if x1=**1** then rain='Annual';

if x2=**1** then rain='Wet';

if x3=**1** then rain='Dry';

drop _group_;

**run**;

**data** Parameterestimates;

set annual_Parameterestimates(in=x1) wet_Parameterestimates(in=x2) dry_Parameterestimates(in=x3);

if x1=**1** then rain='Annual';

if x2=**1** then rain='Wet';

if x3=**1** then rain='Dry';

drop _group_;

**run**;

**data** Smoothedcycle;

set annual_Smoothedcycle(in=x1) wet_Smoothedcycle(in=x2) dry_Smoothedcycle(in=x3);

if x1=**1** then rain='Annual';

if x2=**1** then rain='Wet';

if x3=**1** then rain='Dry';

drop _group_;

**run**;
