# Supplementary material for: Long-term historical and projected herbivore population dynamics in Ngorongoro crater, Tanzania
Source: PLoS One. 2020 Mar 10;15(3):e0212530. doi: 10.1371/journal.pone.0212530 (PMC7064247; doi:10.1371/journal.pone.0212530)
Supplement: S2 Text — (DOCX) [file pone.0212530.s021.docx]

/*-------------------Modelling time trends in population size using semiparametric regression-----*/

/*------Semiparametric regression to reveal the trends----*/;

**Proc** **glimmix** data=NGORONGORO3 Noclprint Noitprint Method=RSPL nofit;

where season='Wet';

by season ;

class species;

Model count= species species*Time /noint dist=negbin link=log ddfm=kr;

Random Time /sub=species type=pspline knotmethod=equal (**20**);

Random Time /sub=intercept type=pspline knotmethod=equal (**20**);

output out=sasuser.Ngorongoro_pred_wet Pred(ilink)=mu LCL(ilink)=Lower UCL(ilink)=Upper;

nloptions tech=NRRIDG Maxiter=**1000** maxfunc=**1000**;

Parms (**0.000040**) (**0.000471**) (**0.2725**);

**run**;

ods output estimates=estimates1 covparms=covparms1;

**Proc** **glimmix** data=NGORONGORO3 Noclprint Noitprint Method=RSPL;

where season='Wet';

by season ;

class group;

effect spl = spline(time);*Constructed spline effects´;

Model count= group group*spl /noint dist=negbin link=log ddfm=kr;

*Random time /sub=group type=pspline knotmethod=equal (20);

*Random time /sub=intercept type=pspline knotmethod=equal (20);

output out=Ngorongoro_pred_wet Pred(ilink)=mu LCL(ilink)=Lower UCL(ilink)=Upper;

nloptions tech=NRRIDG Maxiter=**1000** maxfunc=**1000**;

*Parms (0.000040) (0.000471) (0.2725);

Parms (**0.1951**);

estimate 'Wildebeest at time=1964' group **1** **0** group*spl [**1**,**1** **19.64**] /e;

estimate 'Wildebeest at time=1974' group **1** **0** group*spl [**1**,**1** **19.74**]/e;

estimate 'Wildebeest at time=2011' group **1** **0** group*spl [**1**,**1** **20.116**]/e;

estimate 'Diff for Wildebeest at time= 1964 vs time= 1974' group*spl [-**1**,**1** **19.64**] [**1**,**1** **19.74**],

'Diff for Wildebeest at time= 1974 vs time= 2011' group*spl [-**1**,**1** **19.74**] [**1**,**1** **20.116**]/e adjust=sim(seed=**1**) stepdown;

estimate 'Zebra at time=1964' group **0** **1** group*spl [**1**,**2** **19.64**] /e;

estimate 'Zebra at time=1974' group **0** **1** group*spl [**1**,**2** **19.74**]/e;

estimate 'Zebra at time=2011' group **0** **1** group*spl [**1**,**2** **20.116**]/e;

estimate 'Diff for Zebra at time= 1964 vs time= 1974' group*spl [-**1**,**2** **19.64**] [**1**,**2** **19.74**],

'Diff for Zebra at time= 1974 vs time= 2011' group*spl [-**1**,**2** **19.74**] [**1**,**2** **20.116**]/e adjust=sim(seed=**1**) stepdown;

estimate 'Buffalo at time=1964' group **0** **0** **1** group*spl [**1**,**3** **19.64**] /e;

estimate 'Buffalo at time=1974' group **0** **0** **1** group*spl [**1**,**3** **19.74**]/e;

estimate 'Buffalo at time=2011' group **0** **0** **1** group*spl [**1**,**3** **20.116**]/e;

estimate 'Diff for Buffalo at time= 1964 vs time= 1974' group*spl [-**1**,**3** **19.64**] [**1**,**3** **19.74**],

'Diff for Buffalo at time= 1974 vs time= 2011' group*spl [-**1**,**3** **19.74**] [**1**,**3** **20.116**]/e adjust=sim(seed=**1**) stepdown;

estimate 'Tgazelle at time=1964' group **0** **0** **0** **1** group*spl [**1**,**4** **19.64**] /e;

estimate 'Tgazelle at time=1974' group **0** **0** **0** **1** group*spl [**1**,**4** **19.74**]/e;

estimate 'Tgazelle at time=2011' group **0** **0** **0** **1** group*spl [**1**,**4** **20.116**]/e;

estimate 'Diff for Tgazelle at time= 1964 vs time= 1974' group*spl [-**1**,**4** **19.64**] [**1**,**4** **19.74**],

'Diff for Tgazelle at time= 1974 vs time= 2011' group*spl [-**1**,**4** **19.74**] [**1**,**4** **20.116**]/e adjust=sim(seed=**1**) stepdown;

estimate 'Ggazelle at time=1964' group **0** **0** **0** **0** **1** group*spl [**1**,**5** **19.64**] /e;

estimate 'Ggazelle at time=1974' group **0** **0** **0** **0** **1** group*spl [**1**,**5** **19.74**]/e;

estimate 'Ggazelle at time=2011' group **0** **0** **0** **0** **1** group*spl [**1**,**5** **20.116**]/e;

estimate 'Diff for Ggazelle at time= 1964 vs time= 1974' group*spl [-**1**,**5** **19.64**] [**1**,**5** **19.74**],

'Diff for Ggazelle at time= 1974 vs time= 2011' group*spl [-**1**,**5** **19.74**] [**1**,**5** **20.116**]/e adjust=sim(seed=**1**) stepdown;

estimate 'Elephant at time=1964' group **0** **0** **0** **0** **0** **1** group*spl [**1**,**6** **19.64**] /e;

estimate 'Elephant at time=1974' group **0** **0** **0** **0** **0** **1** group*spl [**1**,**6** **19.74**]/e;

estimate 'Elephant at time=2011' group **0** **0** **0** **0** **0** **1** group*spl [**1**,**6** **20.116**]/e;

estimate 'Diff for Elephant at time= 1964 vs time= 1974' group*spl [-**1**,**6** **19.64**] [**1**,**6** **19.74**],

'Diff for Elephant at time= 1974 vs time= 2011' group*spl [-**1**,**6** **19.74**] [**1**,**6** **20.116**]/e adjust=sim(seed=**1**) stepdown;

estimate 'Blackrhino at time=1964' group **0** **0** **0** **0** **0** **0** **1** group*spl [**1**,**7** **19.64**] /e;

estimate 'Blackrhino at time=1974' group **0** **0** **0** **0** **0** **0** **1** group*spl [**1**,**7** **19.74**]/e;

estimate 'Blackrhino at time=2011' group **0** **0** **0** **0** **0** **0** **1** group*spl [**1**,**7** **20.116**]/e;

estimate 'Diff for Blackrhino at time= 1964 vs time= 1974' group*spl [-**1**,**7** **19.64**] [**1**,**7** **19.74**],

'Diff for Blackrhino at time= 1974 vs time= 2011' group*spl [-**1**,**7** **19.74**] [**1**,**7** **20.116**]/e adjust=sim(seed=**1**) stepdown;

estimate 'Eland at time=1964' group **0** **0** **0** **0** **0** **0** **0** **1** group*spl [**1**,**8** **19.64**] /e;

estimate 'Eland at time=1974' group **0** **0** **0** **0** **0** **0** **0** **1** group*spl [**1**,**8** **19.74**]/e;

estimate 'Eland at time=2011' group **0** **0** **0** **0** **0** **0** **0** **1** group*spl [**1**,**8** **20.116**]/e;

estimate 'Diff for Eland at time= 1964 vs time= 1974' group*spl [-**1**,**8** **19.64**] [**1**,**8** **19.74**],

'Diff for Eland at time= 1974 vs time= 2011' group*spl [-**1**,**8** **19.74**] [**1**,**8** **20.116**]/e adjust=sim(seed=**1**) stepdown;

estimate 'Kongoni at time=1964' group **0** **0** **0** **0** **0** **0** **0** **0** **1** group*spl [**1**,**9** **19.64**] /e;

estimate 'Kongoni at time=1974' group **0** **0** **0** **0** **0** **0** **0** **0** **1** group*spl [**1**,**9** **19.74**]/e;

estimate 'Kongoni at time=2011' group **0** **0** **0** **0** **0** **0** **0** **0** **1** group*spl [**1**,**9** **20.116**]/e;

estimate 'Diff for Kongoni at time= 1964 vs time= 1974' group*spl [-**1**,**9** **19.64**] [**1**,**9** **19.74**],

'Diff for Kongoni at time= 1974 vs time= 2011' group*spl [-**1**,**9** **19.74**] [**1**,**9** **20.116**]/e adjust=sim(seed=**1**) stepdown;

estimate 'Waterbuck at time=1964' group **0** **0** **0** **0** **0** **0** **0** **0** **0** **1** group*spl [**1**,**10** **19.64**] /e;

estimate 'Waterbuck at time=1974' group **0** **0** **0** **0** **0** **0** **0** **0** **0** **1** group*spl [**1**,**10** **19.74**]/e;

estimate 'Waterbuck at time=2011' group **0** **0** **0** **0** **0** **0** **0** **0** **0** **1** group*spl [**1**,**10** **20.116**]/e;

estimate 'Diff for Waterbuck at time= 1964 vs time= 1974' group*spl [-**1**,**10** **19.64**] [**1**,**10** **19.74**],

'Diff for Waterbuck at time= 1974 vs time= 2011' group*spl [-**1**,**10** **19.74**] [**1**,**10** **20.116**]/e adjust=sim(seed=**1**) stepdown;

estimate 'Ostrich at time=1964' group **0** **0** **0** **0** **0** **0** **0** **0** **0** **0** **1** group*spl [**1**,**11** **19.64**] /e;

estimate 'Ostrich at time=1974' group **0** **0** **0** **0** **0** **0** **0** **0** **0** **0** **1** group*spl [**1**,**11** **19.74**]/e;

estimate 'Ostrich at time=2011' group **0** **0** **0** **0** **0** **0** **0** **0** **0** **0** **1** group*spl [**1**,**11** **20.116**]/e;

estimate 'Diff for Wildebeest at time= 1964 vs time= 1974' group*spl [-**1**,**11** **19.64**] [**1**,**11** **19.74**],

'Diff for Wildebeest at time= 1974 vs time= 2011' group*spl [-**1**,**11** **19.74**] [**1**,**11** **20.116**]/e adjust=sim(seed=**1**) stepdown;

estimate 'Warthog at time=1964' group **0** **0** **0** **0** **0** **0** **0** **0** **0** **0** **0** **1** group*spl [**1**,**12** **19.64**] /e;

estimate 'Warthog at time=1974' group **0** **0** **0** **0** **0** **0** **0** **0** **0** **0** **0** **1** group*spl [**1**,**12** **19.74**]/e;

estimate 'Warthog at time=2011' group **0** **0** **0** **0** **0** **0** **0** **0** **0** **0** **0** **1** group*spl [**1**,**12** **20.116**]/e;

estimate 'Diff for Warthog at time= 1964 vs time= 1974' group*spl [-**1**,**12** **19.64**] [**1**,**12** **19.74**],

'Diff for Warthog at time= 1974 vs time= 2011' group*spl [-**1**,**12** **19.74**] [**1**,**12** **20.116**]/e adjust=sim(seed=**1**) stepdown;

**run**;

/*------Modeling cycles in wet season rainfall data--*/;

ods graphics on;

ods output outliersummary=wet_outliersummary ParameterEstimates=wet_ParameterEstimates

ComponentSignificance=wet_ComponentSignificance SmoothedCycle1=wet_SmoothedCycle1 SmoothedCycle2=wet_SmoothedCycle2

SmoothedLevel=wet_smoothedlevel SmoothedTrend=wet_SmoothedTrend;

**proc** **ucm** data=rain4xx;

*id year interval=year;

model wetstd;

irregular;

level plot=smooth checkbreak PRINT=SMOOTH;

slope plot=smooth ;

cycle plot=(filter smooth) PRINT=SMOOTH;

cycle plot=(filter smooth) PRINT=SMOOTH;

*cycle plot=(filter smooth) ;

estimate back=**0** plot=(loess panel cusum wn);

forecast back=**0** lead=**0** plot=(forecasts decomp) PRINT=DECOMP;

**run**;

ods graphics off;

/*-----------------------VARMAX(2,2,0) model for wildebeest----------------------------------*/;

ods graphics on;

**proc** **varmax** data=data.Scenarios_rain10 plots=all;

by Scenario;

*where Season="Wet";

*id date interval=semiyear align=end;

ods output DiagnostAR=WL_DiagnostAR DiagnostWN=WL_DiagnostWN ANOVA=WL_ANOVA MARoots=WL_MARoots ARRoots=WL_ARRoots

PortmanteauTest=WL_PortmanteauTest;

nloptions tech=newrap maxit=**1000** pall;

model wildebeest_dry Wildebeest_wet

=dry wet /p=**2** q=**2** xlag=(**1**,**2**,**3**,**4**,**5**) /*nocurrentx*/

printform=univariate minic=(type=aic p=**4**)

print=(corry corrx pcorr pcancorr parcoef Estimates roots diagnose) method=ml;

*Restrict XL1_1_1=0, XL1_1_2 =0, XL2_1_1=0, XL2_1_2=0, XL3_1_1=0, XL3_1_2=0;

*GARCH Q=2 form=BEKK;

*cointeg rank=1 ectrend /*exogeneity*/;

ods output corrxgraph=WL_corrxgraph corrygraph=WL_corrygraph CorrXLags=WL_CorrXLags

CorrXbyVar=WL_CorrXbyVar CorrYLags=WL_CorrYLags CorrYbyVar=WL_CorrYbyVar

PartialAR=PartialAR ParameterEstimates=WL_ParameterEstimates ParameterGraph=WL_ParameterGraph

PartialAR=WL_PartialAR PartialARGraph=WL_PartialARGraph PartialCanCorr=WL_PartialCanCorr

PartialCorr=WL_PartialCorr PartialCorrbyVar=WL_PartialCorrbyVar PartialCorrGraph=WL_PartialCorrGraph;

output out=WL_pred_dry_Wet_wild lead=**88** back=**0**;

**run**;

ods graphics off;
