# Supplementary figures and images for: Long-term historical and projected herbivore population dynamics in Ngorongoro crater, Tanzania
Source: PLoS One. 2020 Mar 10;15(3):e0212530. doi: 10.1371/journal.pone.0212530 (PMC7064247; doi:10.1371/journal.pone.0212530)

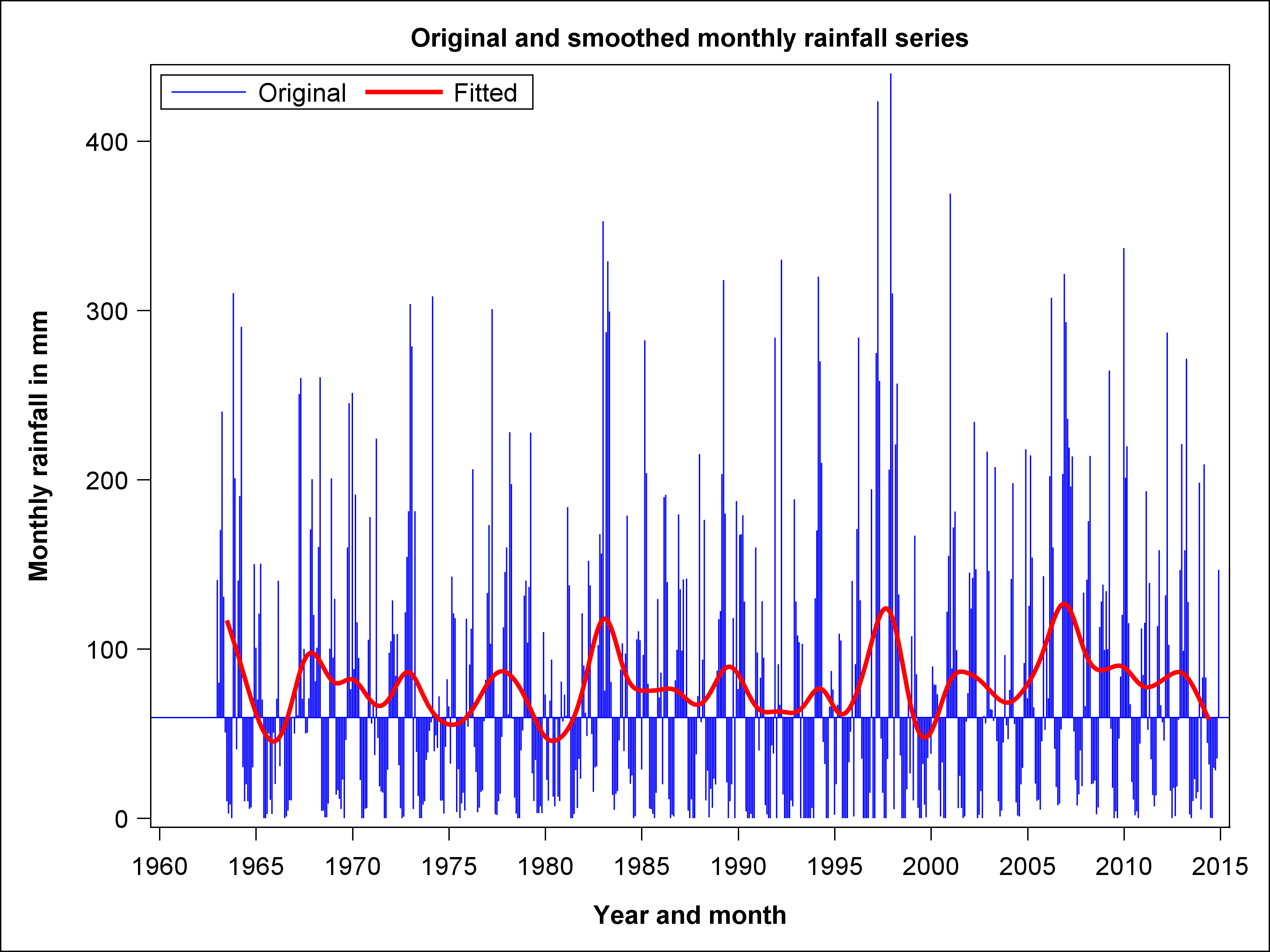

Supplement: S1 Fig — (PNG) [file pone.0212530.s022.png]

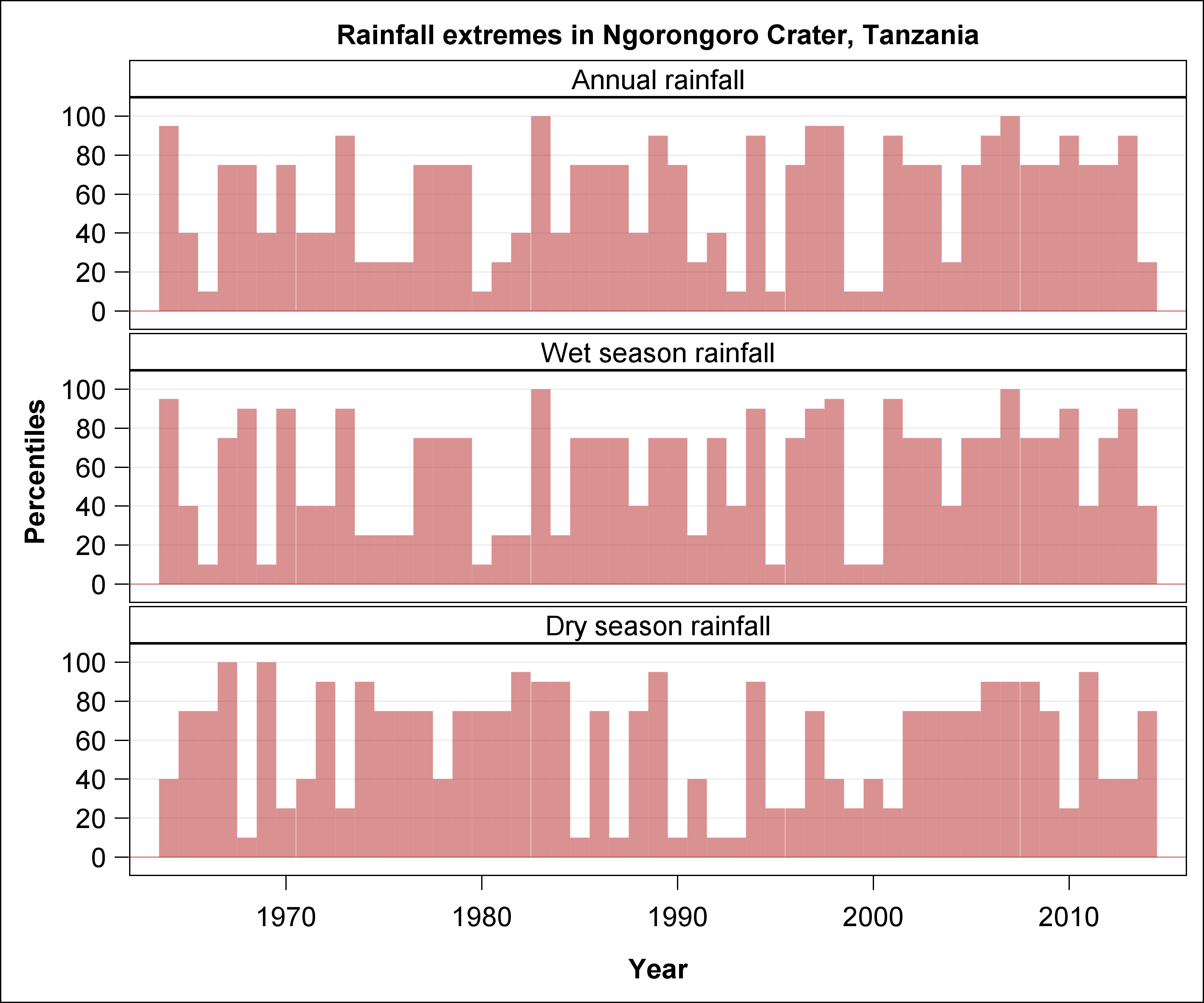

Supplement: S2 Fig — The percentiles are used to classify years or seasons as extreme, severe or moderate drought years or seasons, normal, wet, very wet or extremely wet years or seasons as described in the text. (PNG) [file pone.0212530.s023.png]

**Spectrum of Annual rainfall**

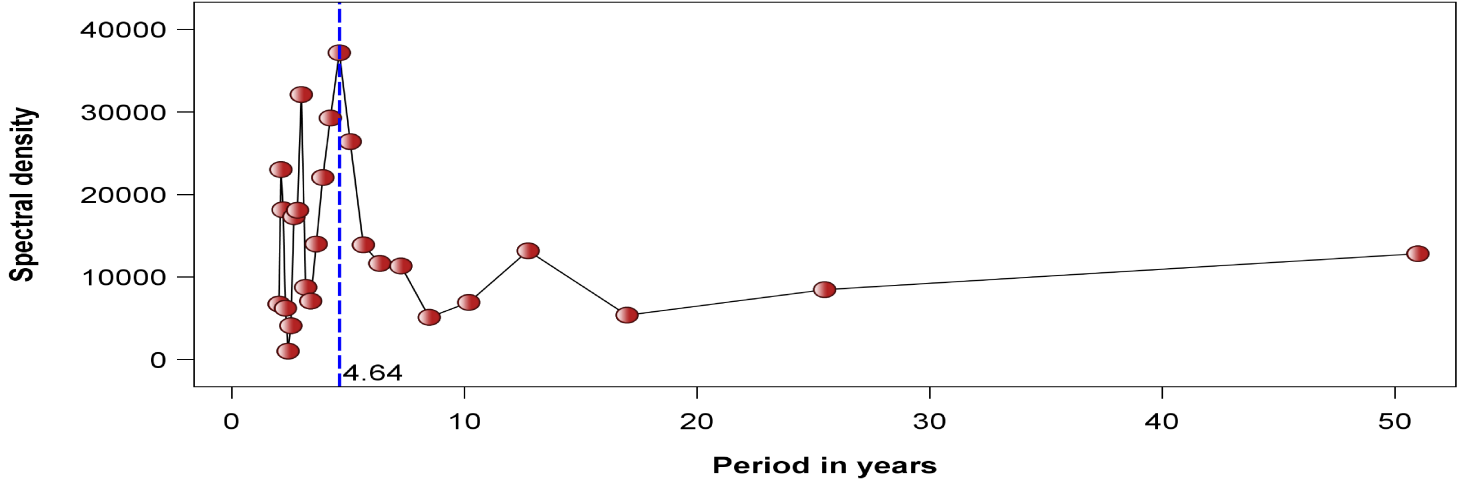

**Spectrum of wet season rainfall**

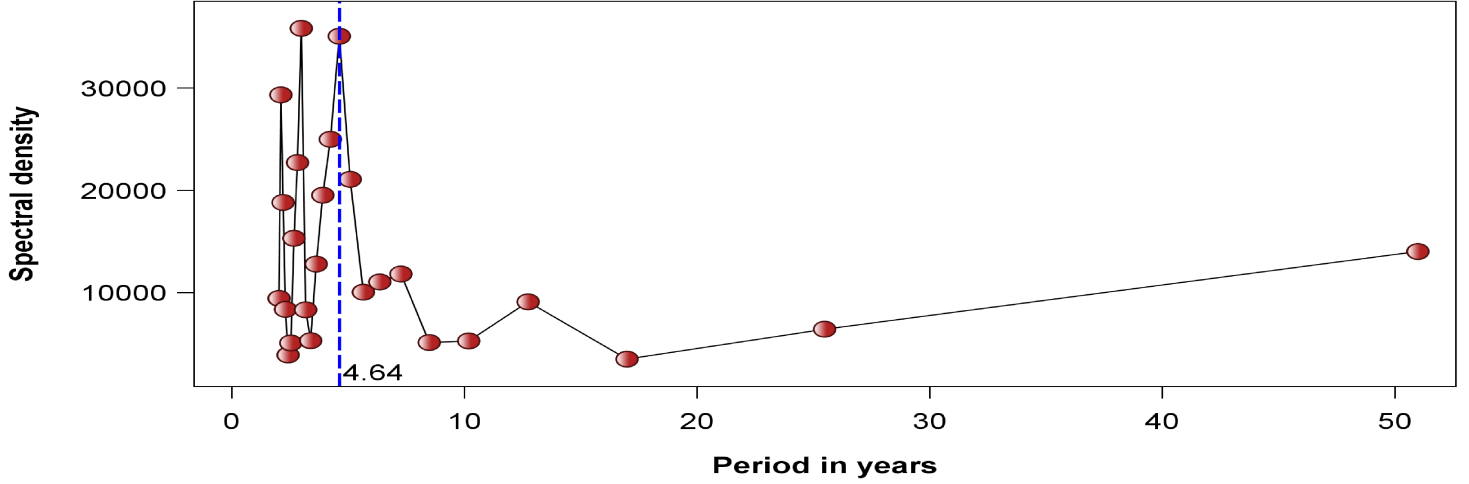

**Spectrum of Dry season rainfall**

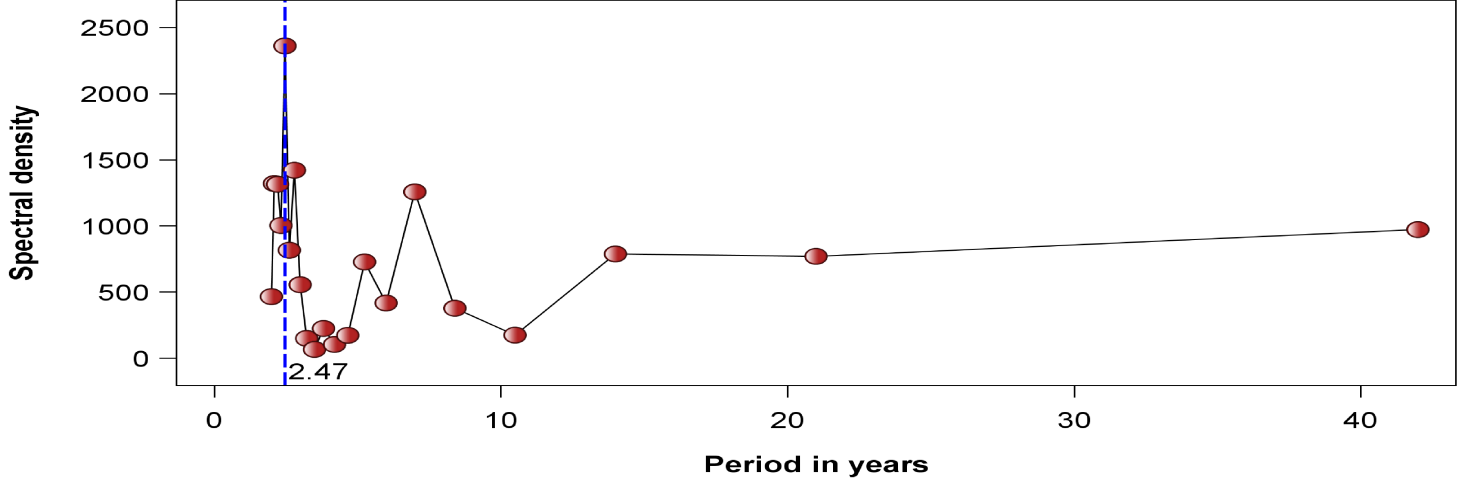

Supplement: S3 Fig — Spectral density versus period of cycles (in years) for a) annual rainfall, b) wet season rainfall, and c) dry season rainfall based on rainfall recorded at the Ngorongoro Conservation Authority Headquarters from 1963 to 2014. A large value of spectral density means that the corresponding cycle period has greater support in the data. (PDF) [file pone.0212530.s024.pdf]

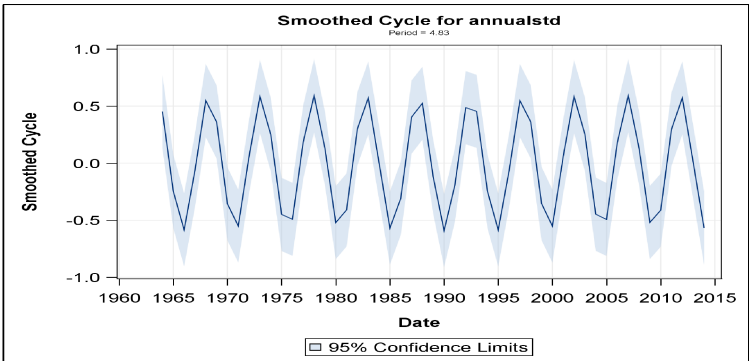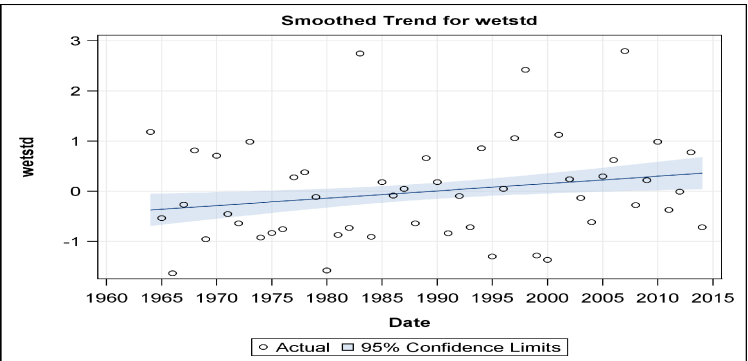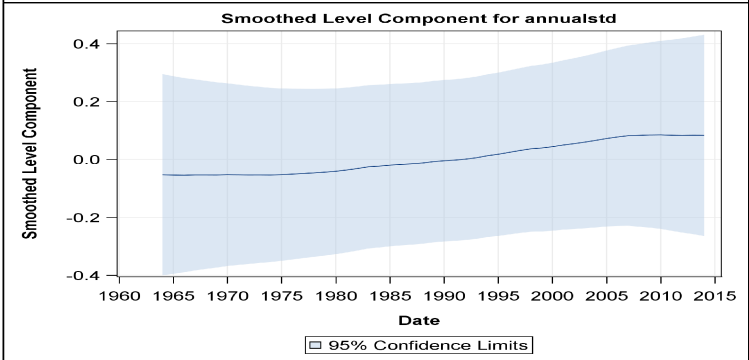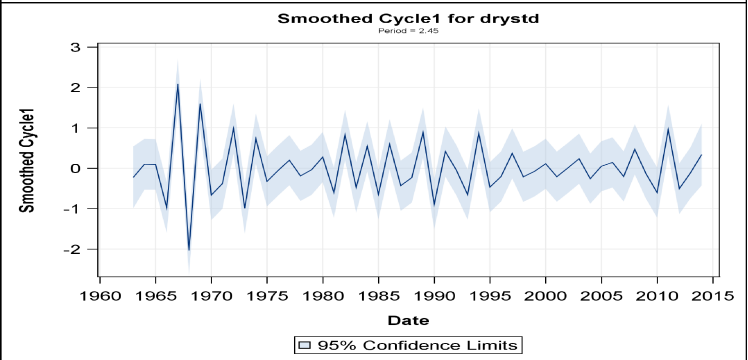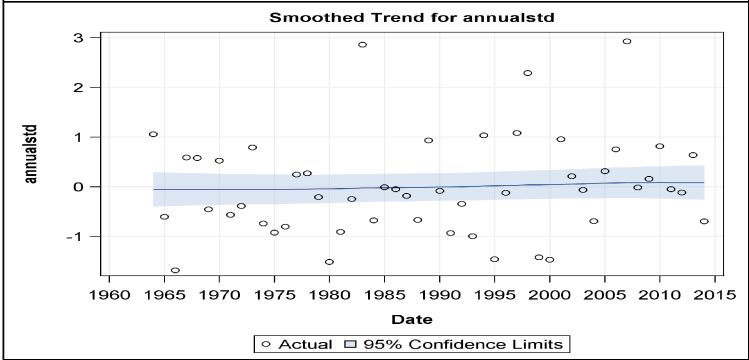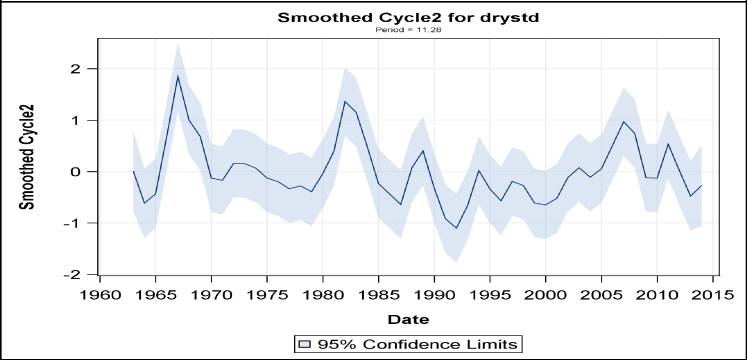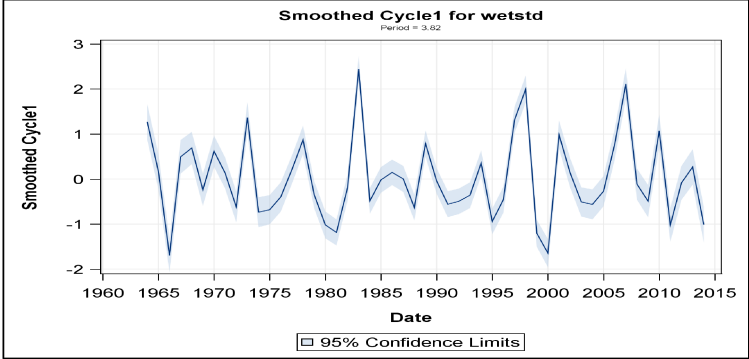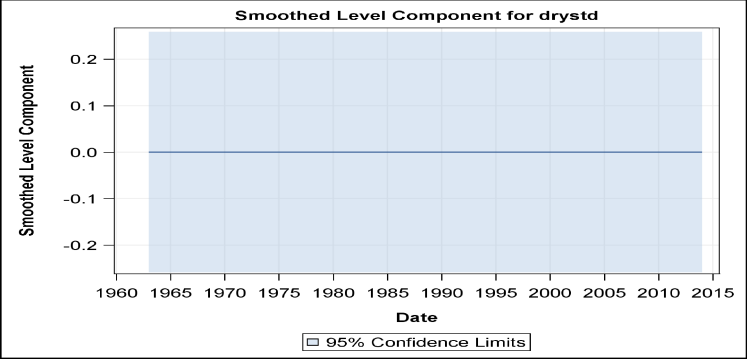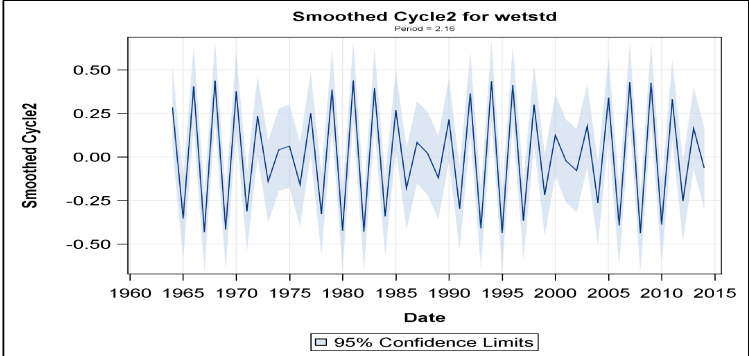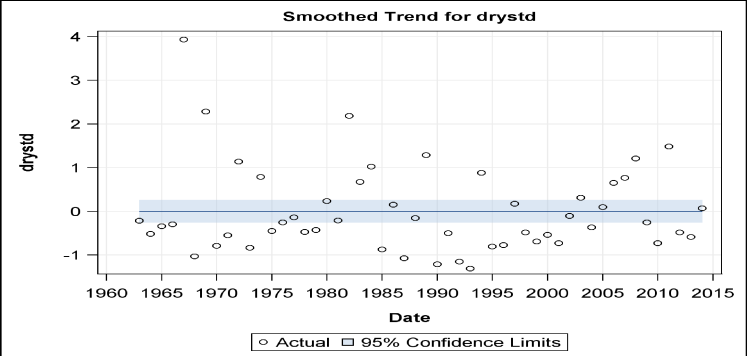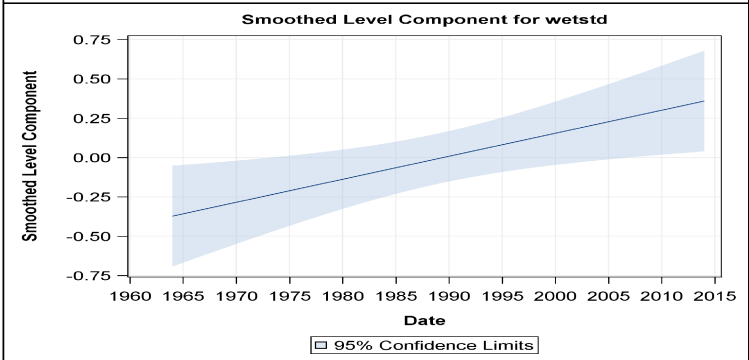

Supplement: S4 Fig — (PDF) [file pone.0212530.s025.pdf]

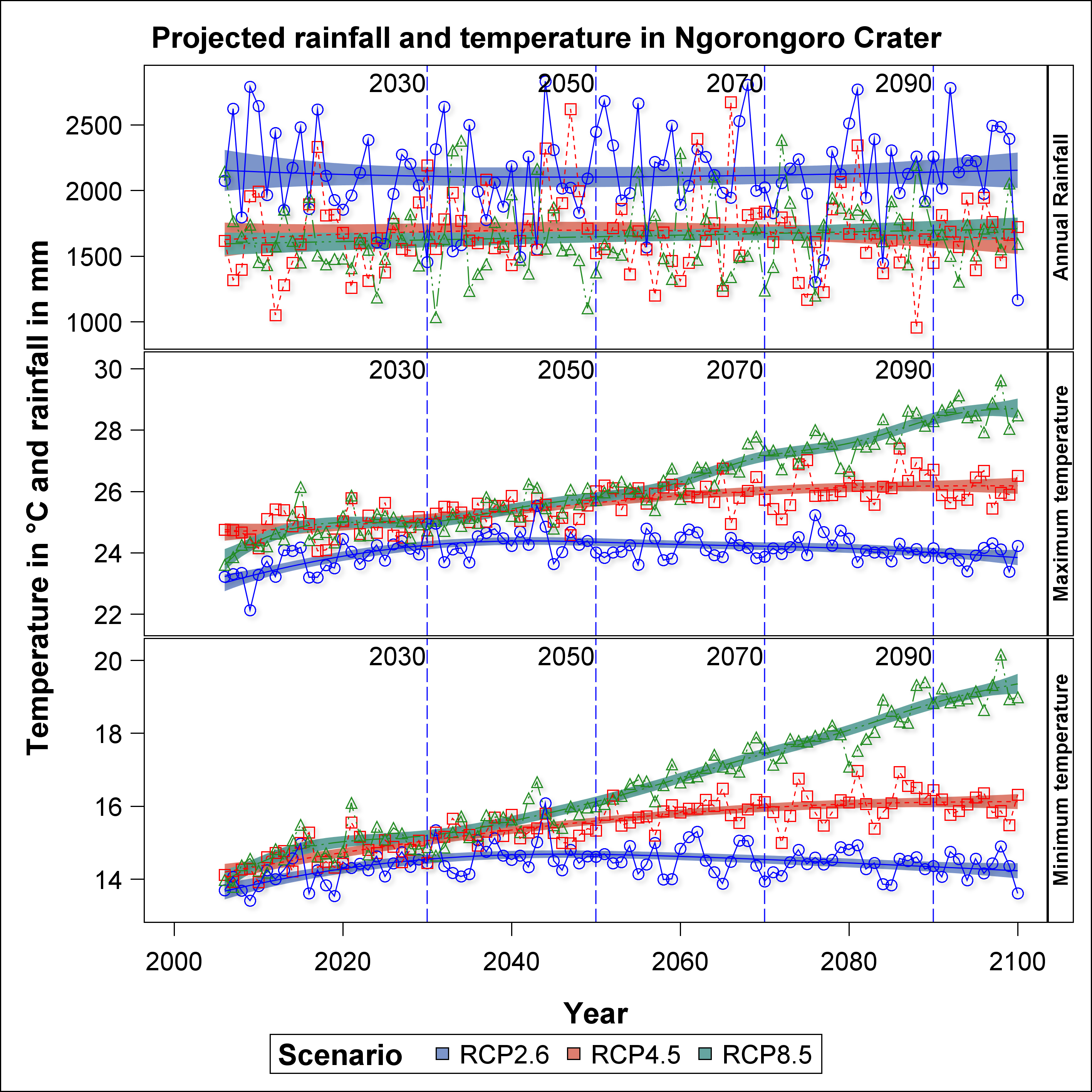

Supplement: S5 Fig — (PNG) [file pone.0212530.s026.png]

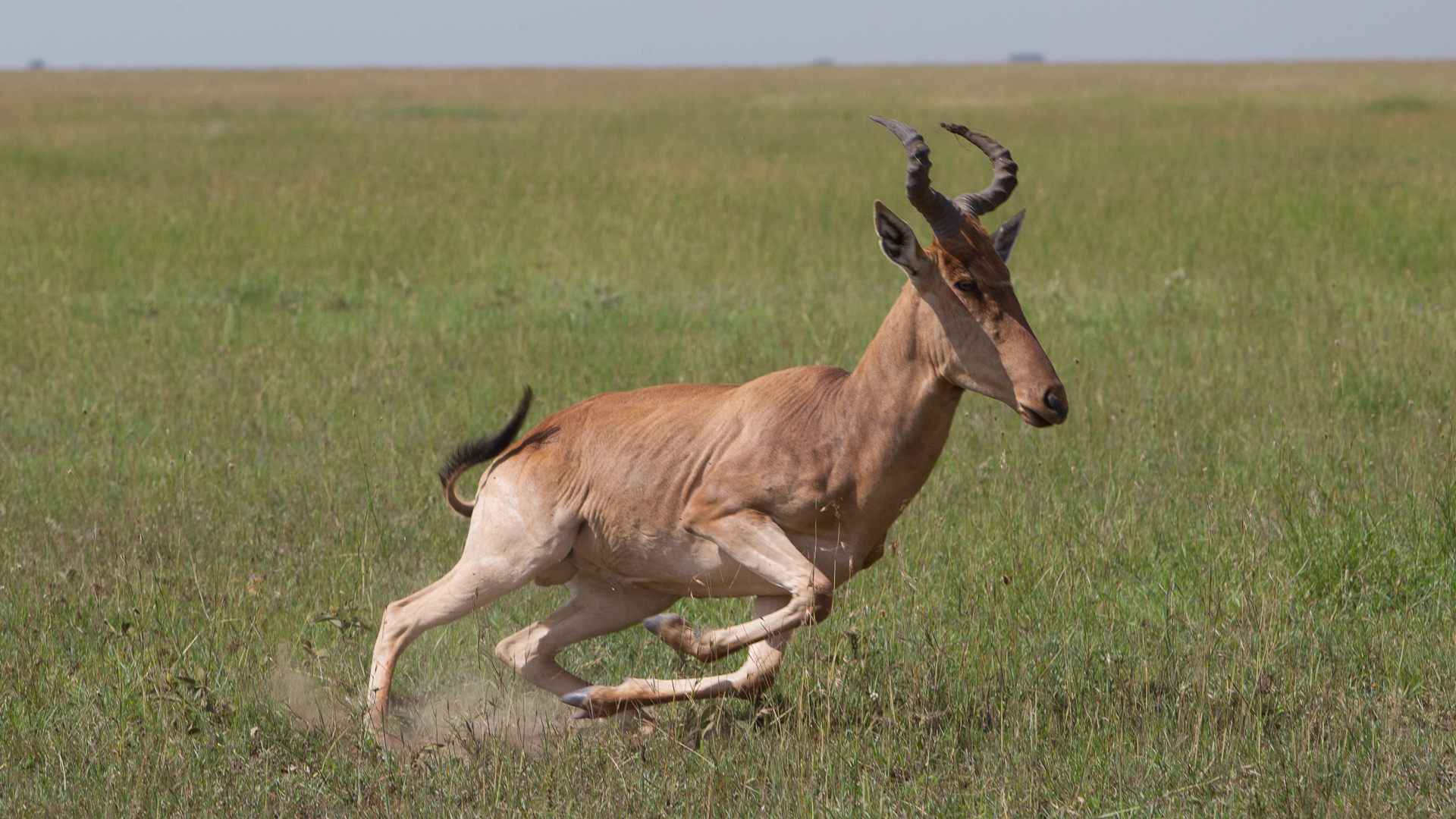

Supplement: S1 File — (JPG) [file pone.0212530.s027.jpg]
